# Unleashing the Potential of MXene‐Based Flexible Materials for High‐Performance Energy Storage Devices

**DOI:** 10.1002/advs.202304874

**Published:** 2023-11-08

**Authors:** Yunlei Zhou, Liting Yin, Shuangfei Xiang, Sheng Yu, Hannah M. Johnson, Shaolei Wang, Junyi Yin, Jie Zhao, Yang Luo, Paul K. Chu

**Affiliations:** ^1^ Hangzhou Institute of Technology Xidian University Hangzhou 311200 China; ^2^ School of Mechano‐Electronic Engineering Xidian University Xi'an 710071 China; ^3^ Department of Aerospace and Mechanical Engineering University of Southern California Los Angeles CA 90089 USA; ^4^ School of Materials Science and Engineering and Institute of Smart Fiber Materials Zhejiang Sci‐Tech University Hangzhou 310018 China; ^5^ Department of Chemistry Washington State University Pullman WA 99164 USA; ^6^ Department of Bioengineering University of California Los Angeles Los Angeles CA 90095 USA; ^7^ Molecular Engineering of Polymers Department of Material Science Fudan University Shanghai 200438 China; ^8^ Department of Materials ETH Zurich Zurich 8093 Switzerland; ^9^ Department of Physics Department of Materials Science and Engineering and Department of Biomedical Engineering City University of Hong Kong Kowloon Hong Kong 999077 China

**Keywords:** batteries, energy storage, flexible materials, MXenes, supercapacitors

## Abstract

Since the initial discovery of Ti_3_C_2_ a decade ago, there has been a significant surge of interest in 2D MXenes and MXene‐based composites. This can be attributed to the remarkable intrinsic properties exhibited by MXenes, including metallic conductivity, abundant functional groups, unique layered microstructure, and the ability to control interlayer spacing. These properties contribute to the exceptional electrical and mechanical performance of MXenes, rendering them highly suitable for implementation as candidate materials in flexible and wearable energy storage devices. Recently, a substantial number of novel research has been dedicated to exploring MXene‐based flexible materials with diverse functionalities and specifically designed structures, aiming to enhance the efficiency of energy storage systems. In this review, a comprehensive overview of the synthesis and fabrication strategies employed in the development of these diverse MXene‐based materials is provided. Furthermore, an in‐depth analysis of the energy storage applications exhibited by these innovative flexible materials, encompassing supercapacitors, Li‐ion batteries, Li–S batteries, and other potential avenues, is conducted. In addition to presenting the current state of the field, the challenges encountered in the implementation of MXene‐based flexible materials are also highlighted and insights are provided into future research directions and prospects.

## Graphical abstract

This paper delves into MXene materials' synthesis methods and their versatile applications in energy storage, spanning supercapacitors, Li‐ion, and Li–S batteries, discovering the current landscape, hurdles, and exciting research opportunities in this dynamic field.

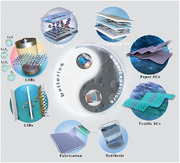

## 1 Introduction

Energy generation and consumption is a central societal issue, impacting our way of life, world economy, environment, and human health.^[^ ^1^, ^2^ ^]^ Green and sustainable energy resources such as wind energy and solar energy are critical when considering the impacts of climate change; however, they are also naturally intermittent sources, and therefore effective energy storage technologies are imperative for consistent power storage and release.^[^ ^3^, ^4^, ^5^ ^]^ Electrochemical energy storage devices have already been extensively developed for use in electric vehicles, consumer electronics, and energy storage grids and offer properties such as a wide working range, large power and energy density, and high conversion efficiency.^[^ ^6^, ^7^, ^8^ ^]^ However, their performances are still not sufficient to meet the fast‐growing demands of large‐scale energy storage applications. Moreover, next‐generation wearable and portable devices that also require energy storage components are being developed for multifunctional and intimate integration with the human body.^[^ ^9^, ^10^ ^]^ These applications necessitate rigorous requirements to not only maintain high electrochemical performances, but also possess excellent flexibility and conformation freedom. The aforementioned problems motivate the development of advanced materials that can successfully balance enhanced energy and power density, superior physical and chemical performances, and advantageous flexibility.

The emerging carbides, nitrides, and carbonitrides of 2D transition metals known as MXenes have seen significant research growth in recent years.^[^ ^11^, ^12^, ^13^ ^]^ Thanks to their superior properties such as microscale lateral size, atomic layer thickness, and prominent hydrophilicity, MXenes can be easily assembled into flexible film/paper electrodes by vacuum‐assisted filtration or coating techniques.^[^ ^14^, ^15^, ^16^ ^]^ Additionally, the abundant surface chemistry as well as the high electronegativity imparted by hydrofluoric acid‐based etching makes it possible for MXenes to combine with other nanomaterials to prepare high‐performance flexible composite electrodes.^[^ ^17^ ^]^ MXene‐based flexible materials illustrate astonishing conductivity and extraordinary flexibility; their high electrical conductivity facilitates fast electron transport, while their unique lamellar structure provides a low diffusion barrier for ion expansion.^[^ ^18^, ^19^ ^]^ Concomitantly, the ability to withstand large amounts of strain provides MXene‐based flexible materials the capacity to ameliorate cyclic performances and integrate into flexible energy systems.^[^ ^20^, ^21^ ^]^ Benefiting from these fascinating advantages, MXene and MXene‐based flexible materials have been elaborately designed for multiple applications in both batteries that require the high energy density and various supercapacitors (SCs) with the fast charge–discharge process.^[^ ^22^, ^23^, ^24^, ^25^, ^26^ ^]^

**Figure 1:**
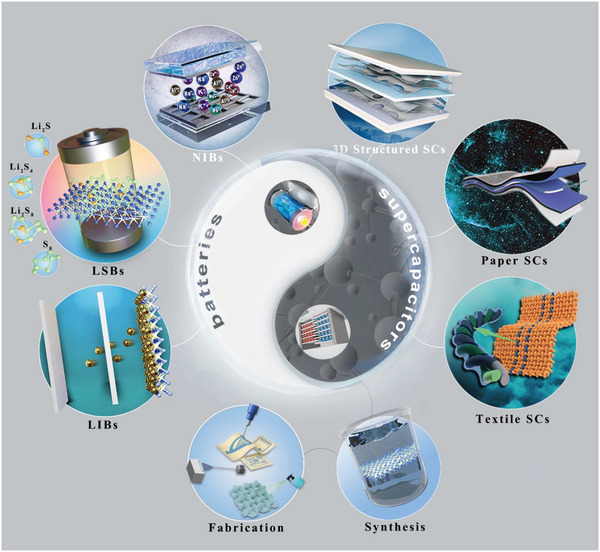
Schematic of MXene‐based materials for energy storage applications. Reproduced with permission.^[^ ^31^, ^32^, ^33^, ^34^, ^35^ ^]^ Copyright 2018, Wiley‐VCH; Copyright 2019, Nature Publishing Group; Copyright 2021, The Royal Society of Chemistry.

Previous review articles have indeed delved into the realm of flexible MXene‐based batteries and supercapacitors.^[^ ^18^, ^27^ ^]^ Nonetheless, these reviews primarily focused their attention on the methodologies associated with the synthesis of MXene nanoflakes, the inherent attributes of MXene monomers, and their utilization within a limited spectrum of energy storage devices. Regrettably, only a sparse few of these reviews presented a comprehensive overview of the broader landscape encompassing MXene‐based flexible materials and their potential in the context of energy storage.^[^ ^20^, ^28^, ^29^, ^30^ ^]^ This underscores the pressing need to advance our comprehension of MXene‐based flexible materials and their extensive range of applications in batteries and supercapacitors. In this review, we mainly summarize the impact of synthesis and fabrication strategies for MXene and MXene‐based flexible materials, the accessible technologies for constructing their strain‐withstanding ability, as well as their specific applications and advantages in batteries and SCs (**Figure** **1**). Finally, the current challenges and future prospects for MXene‐based energy electronics are briefly discussed.

## 2 Synthesis and Fabrication of MXenes

### 2.1 Synthesis Strategies of MXenes

MXenes are so named because they are constructed from early transition metals (M = Ti, V, Cr, Nb, etc.) and carbon and/or nitrogen (X = C or N), while the ene suffix refers to their structural similarity to 2D graphene.^[^ ^36^ ^]^ The specific synthetic method employed to generate MXene materials has a direct influence on their interlayer structure and terminal groups, further imparting effects on the performances of the resulting energy storage devices. The synthetic methods of MXene nanosheets can be categorized into two broad strategies: top‐down preparations (focused on subtractive manipulations of bulk materials) and bottom‐up preparations (focused on the assembly of subcomponents).

#### 2.1.1 Top‐Down Synthetic Strategies

First, the top‐down synthesis strategy refers to selectively removing the “A” layers (A = element such as Al, Si, Sn, In, etc.) of an MAX precursor and then exfoliating. In contrast with other layered materials such as graphite and transition metal dichalcogenides (TMDs) that possess bonding between 2D layers by van der Waals interactions, M–A bonding is metallic in character and is difficult to remove with mechanical methods. However, the metallic bond between M–A is more chemically active than M–X, which instead enables selective chemical etching of A layers. Aqueous hydrofluoric acid (HF) etching has been the earliest and most commonly used way to remove Al‐atom layer from MAX phases and prepare MXenes.^[^ ^37^ ^]^ Taking Ti_3_AlC_2_ MAX material as an example, the chemical reactions of HF etching and MXene synthesis can be written as

(1) $$2{\mathrm{Ti}}_{3}{\mathrm{AlC}}_{2}+6\mathrm{HF}\to 2{\mathrm{AlF}}_{3}+3H_{2}+2{\mathrm{Ti}}_{3}C_{2}$$

(2) $${\mathrm{Ti}}_{3}C_{2}+2H_{2}O\to {\mathrm{Ti}}_{3}C_{3}{\left(\mathrm{OH}\right)}_{2}+H_{2}$$

(3) $${\mathrm{Ti}}_{3}C_{2}+2\mathrm{HF}\to {\mathrm{Ti}}_{3}C_{2}F_{2}+H_{2}$$

These equations illustrate the gradual etching process of MXene nanomaterials. The Al layer of precursor is removed in Equation (1), followed by the simultaneous reactions depicted in Equations (2) and (3), producing an M*_n_*_+1_X*_n_* T*_x_* material with ─F, ─OH, and ─O functional groups. Furthermore, the HF etching of other A elements is also accessible, such as removing Si atoms from the Ti_3_SiC_2_ MAX phase.^[^ ^38^ ^]^ It is worth noting that the concentration of HF in the solution is pivotal to the successful etching of A‐element layers. A low HF content will lead to only partially etched A layers, while HF content that is too high will cause excessive etching which may destroy or completely dissolve MAX materials.

Despite its efficacy as an etchant, HF is environmentally toxic and corrosive, which causes danger in experimental utilization and is prohibited in many research groups. Thus, some studies choose substituted solutions that can generate HF in situ, such as the mixed solution of hydrochloric acid (HCl) and LiF, NH_4_HF_2_ solution, and 1‐ethyl‐3‐methylimidazolium tetrafluoroborate (EMIMBF_4_).^[^ ^39^, ^40^, ^41^ ^]^ The in situ release of HF provides a safer and milder etching environment of A elements and contributes to the production of MXene flakes with larger lateral size and fewer nanometer‐scale defects, which express clay‐like physical appearance.^[^ ^42^, ^43^ ^]^ Moreover, the alternative F‐containing etching methods generate fewer ─F terminals and provide other surface functional groups instead. For example, the hydrated cations [K(H_2_O)*_x_*)]^+^ in the mixed solution of KF and HCl are affinitive with MXene nanosheets and can be absorbed on their surface.^[^ ^44^ ^]^ And terminal groups like ─Cl possess larger size than ─F, which in turn supplies more interlayer spaces and extra reaction site, ultimately demonstrating better capacitance performances. This is in contrast to the single F‐containing etching process, which can only produce accordion‐like multilayered MXene flakes. The limited interlayer space and surface area constrain the number of available bonding sites, resulting in a restriction of the total energy storage capacity. In order to enhance interlayer space and obtain few/single‐layered MXene dispersions, an effective remedy is to introduce bulky intercalating agents via ultrasonic treatment and repeated washing. In recent studies, commonly used intercalants include organic solvent molecules, such as dimethyl sulfoxide (DMSO), *N*,*N*‐dimethylformamide (DMF), isopropylamine (i‐PrA), and tetrapropylammonium hydroxide (TPAOH), as well as metal cations such as Li^+^, Na^+^, K^+^, Ca^2+^, Mn^2+^, and Al^3+^.^[^ ^45^, ^46^ ^]^ It is noticeable that intercalants demonstrate selectivity and an impart various effects on different MXene materials. For example, DMSO only possesses a delamination effect on Ti_3_C_2_ MXene, while i‐PrA and TPAOH are more universal for different MXene types. Another representative case is that the relatively small size of metal cations typically restrict them from completely delaminating MXenes. Notably, an ultrasonic‐free method known as minimally intensive layer delamination can achieve etching and delamination processes in one step and ensure controllable production of MXene flakes with regulated lateral size and excellent preparation quality by employing the mixed solution of HCl and metal fluoride in suitably high concentrations (e.g., 12 m LiF and 9 m HCl).^[^ ^47^, ^48^ ^]^ Etching and delamination methods mentioned above also present opportunities for the scale‐up of MXene synthesis, which are from the laboratory level to the industry scale.^[^ ^49^ ^]^

Considering that the existence of ─F terminal groups can impede the transportation of intercalants, novel fluoride‐free protocols were studied and reported. Recently, Shi et al. exploited an iodine (I_2_) solution at 100 °C for etching Ti_3_AlC_2_.^[^ ^50^ ^]^ The molar ratio of Ti_3_AlC_2_ powders and I_2_ was 1:3, resulting in more than 70% Ti_3_C_2_T*_x_* with thickness less than 5 nm. Successful preparation of Ti_3_C_2_T*_x_* was also observed in electrochemical etching with HCl alone or in acidic aqueous solution, which resulted in few‐layered MXene nanosheets with a high yield of 90% and a large lateral size of ≈10 µm.^[^ ^51^, ^52^ ^]^ In addition, an NaOH‐assisted hydrothermal alkali treatment method could produce Ti_3_C_2_T*_x_* at 270 °C.^[^ ^53^ ^]^ The synergy of a high temperature and a high NaOH content up to 27.5 m not only ensured the complete etching of Al layer, but also prevented Ti oxidation, which generates harmful Na/K–Ti–O compounds on the surface of MXene, ultimately achieving a high purity up to 92 wt% of multilayered Ti_3_C_2_T*_x_*. Replacing Al^3+^ by other metal cations in ultrahigh‐temperature Lewis acidic molten salts (like ZnCl_2_ and CuCl_2_) was another commonly employed fluoride‐free etching method, and the resulting terminal groups were found to be single‐typed and controllable.^[^ ^42^, ^54^, ^55^ ^]^ Recently, Sun and co‐workers attempted a thermal reduction method to etch S‐containing MAX phases to synthesize MXenes.^[^ ^56^ ^]^ When the Ti_2_SC MAX precursor was heated to a temperature of 800 °C in a hydrogen atmosphere (5% H_2_ in Ar), sulfur was thermally reduced by H_2_ and removed as a volatile gas, resulting in the production of Ti_2_C materials with excellent capacitance and rate performance.

#### 2.1.2 Bottom‐Up Synthetic Strategies

In contrast from the top‐down approach that utilizes subtractive manufacturing, the bottom‐up strategy employs small molecules for the additive growth of directional 2D MXene thin films on substrates. Chemical vapor deposition (CVD) methods have been exploited to grow Mo_2_C MXene crystals on Cu/Mo substrate.^[^ ^57^ ^]^ With a high temperature over 1085 °C, the obtained crystals demonstrated an ultrathin thickness of several nm and an expanded lateral size of ≈100 µm. Additionally, Zhang et al. developed a plasma‐enhanced pulsed‐laser deposition technique to acquire the structure‐controlled growth of MoC_2_ by regulating the crystal orientation of sapphire substrates, which was accomplished at a relatively low temperature of 700 °C.^[^ ^58^ ^]^ MXene crystals grown through bottom‐up strategy have been found to possess significantly larger 2D sizes, fewer defects, and better controllability, but only few‐layered MXenes were observed instead of ideal single‐layered ones. Moreover, the expansive equipment costs and the precise reaction conditions tremendously limit the development of those methods. At present, reports of this strategy are very few and have developed slowly, urging more exploration and investigation in this area.

### 2.2 Fabrication Methods for MXene‐Based Flexible Materials

After being successfully synthesized, MXene dispersions require further fabrication into hierarchical materials, such as textiles, thin films, and porous structured foams, for integration into energy storage applications. The laminated MXene films are laterally bridged through capillary force, which enables their strain‐withstand ability. Due to the mechanical interlocking of doped components, the introduction of polymers (such as polyvinyl alcohol (PVA), poly(3,4‐ethylenedioxythiophene), polystyrene sulfonate (PEDOT:PSS), and polyaniline) as well as low‐dimensional nanomaterials (like nanocellulose) into MXene‐based materials can further improve their mechanical strength and toughness, enhancing ultimate tensile strain. Meanwhile, the flexibility of MXenes and MXene‐based composites can be enhanced by changing their physical morphology to porous 3D structures and textiles. In this section, we will introduce a variety of fabrication methods and their utilization to generate MXene‐based flexible materials. Their adaptability, advantages, and disadvantages are summarized in **Table** **1**.

**Table 1:** The summary of different fabrication strategies.

Fabrication strategy	Fabrication method	Material shape	Flexibility (bending/compression/stretching)	Electrochemical characteristics	Stability	Other limitations	Other adaptability	Refs.
Vacuum filtration		Film	Bending/folding	Limited capacity	Stable for cycling and deformation	Limited interlayer space	Suitable for further process	[65]
Coating	Spin‐coating	Thin film	Bending/folding	Limited cyclic performance	Not very stable	Limited loading of MXenes	Transparent devices	[66]
	Blade‐casting	Film/textile	Bending/folding				Adjustable thickness/vertical‐aligned structure	[67]
	Dip‐coating/drop‐casting/spray‐coating	Fabric/film/foam	Bending/folding/compression/stretching				Combined with templates	[43]
Spinning	Wet spinning	Fiber	Bending/stretching	High conductivity	Stable for cycling and deformation	Need special spinnable solutions	Wearble electronics	[62]
	Electrospinning	Nonwoven textile	Bending	Limited conductivity				[68]
Biscrolling		Fiber	Bending/Folding	High conductivity and capacity	Stable for cycling and deformation	Limited length	Wearable electronics	[31]
Printing		Film	Bending	High conductivity	Not very stable	Need substrate; limited flexibility	Transparent devices; multifuctional and industrial production; patterns	[32]
Template		Fabric/film/foam	Bending/compression/stretching	In dependence of templates and specific methods	In dependence of templates and specific methods	Combine with other methods	Provide extra properties	[69]

#### 2.2.1 Vacuum Filtration Methods

**Figure 2:**
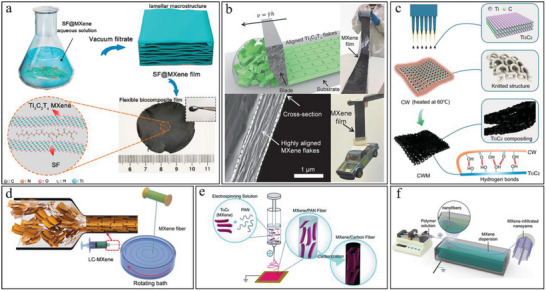
Strategies for fabricating MXene‐based flexible materials. a) Scheme of the vacuum‐assisted filtration for SF@MXene films. Reproduced with permission.^[^ ^59^ ^]^ Copyright 2020, Elsevier. b) Schematic diagram of the blade‐casted MXene films for large‐scale fabrication and the SEM cross‐sectional image. Reproduced with permission.^[^ ^60^ ^]^ Copyright 2020, Wiley‐VCH. c) The preparation process of the MXene/clean wiper (CWM) electrodes via dip‐coating method. Reproduced with permission.^[^ ^61^ ^]^ Copyright 2021, Elsevier. d) Diagram of producing pristine MXene fibers by wet spinning. Reproduced with permission.^[^ ^62^ ^]^ Copyright 2020, American Chemical Society. e) Illustration of the production for MXene/PAN fibers via electrospinning. Reproduced with permission.^[^ ^63^ ^]^ Copyright 2019, The Royal Society of Chemistry. f) A spinning method that employs MXene dispersion as the collector and bath for the combined electrospinning and wet spinning. Reproduced with permission.^[^ ^64^ ^]^ Copyright 2020, Wiley‐VCH.

Vacuum filtration, in which water is removed by vacuum‐assisted filtering through a polypropylene membrane, is generally used to prepare MXene‐based flexible films. When using the vacuum filtration process, the hydrogen bonding between MXene flakes and additives was enhanced, leading to the compact and efficient combination between different components in the resulting flexible films (**Figure** **2**a).^[^ ^70^, ^71^ ^]^ These freestanding films expressed strong mechanical performance and flexibility to resist bending and folding deformation and was available for batteries and supercapacitors.^[^ ^65^ ^]^ Additionally, the tough nature of MXene thin films developed in this manner provide them with the ability to be further processed into 3D structured MXene materials.^[^ ^72^ ^]^ This self‐assembly potential was demonstrated with black phosphorous (BP) through layer‐by‐layer (LbL) filtration, which provided the extra interlayer space and ensured flexibility to prevent MXenes from self‐stacking.^[^ ^73^ ^]^

#### 2.2.2 Coating Methods

Coating methods refer to attaching MXene‐based dispersions onto a variety of flexible substrates and templates which can have different functional properties. Obviously, the coatings deposited by this method are completely conformal with the shape of substrates, and the electrostatic assembly between the surfaces of MXene‐based materials and substrates ensures the deformability of an MXene coating along with its flexible substrate. By means of high‐speed spin‐coating up to hundreds or thousands of rpm, an ultrathin MXene coating was formed and exhibited a uniform thickness and high transmittance, demonstrating its promise for flexible and transparent energy storage devices such as solar cells.^[^ ^66^ ^]^ Another coating method known as blade‐casting is suitable for preparing MXene films with the thickness from sub‐micrometers to tens of micrometers by adjusting the height of blader and the concentration of solution.^[^ ^60^ ^]^ This method illustrates applicability for large‐scale fabrication, and the blade‐induced shear force promoted the generation of highly aligned MXenes with guaranteed strength and toughness (Figure 2b). In addition, blade‐casting was also adapted to coat MXene materials onto fine and closely woven textiles, showing a possibility for fabric energy storage electronics.^[^ ^67^ ^]^

Alternatively, dip‐coating, drop‐casting, and spray‐coating are more common methods for adhering MXene coatings onto template materials, including fibers, yarns, knitted fabrics, textiles, and sponges (Figure 2c).^[^ ^33^, ^61^, ^74^, ^75^, ^76^, ^77^ ^]^ In particular, Gogotsi's group employed a two‐step dip‐coating method.^[^ ^78^ ^]^ Nanosized MXene flakes were first infiltrated into cotton fibers and the large‐sized MXene flakes were then coated on the surface of yarns. The rough surface of cotton fibers contributed to the adhesion of small MXene nanoparticles on them, and the resulting MXene‐coated yarns illustrated an improved energy storage performance while retaining flexibility and high integrability. Relying on the interlayer electrostatic effect, an LbL spraying protocol was presented to strengthen the adhesion between negative MXene coating and positive substrate material as well as decrease the layered distance of MXene, ensuring a coating with the ability to withstand extreme mechanical deformations.^[^ ^43^ ^]^

#### 2.2.3 Spinning Methods

Spinning techniques, including wet spinning and electrospinning, are an advanced strategy to obtain flexible fibers and textiles with high MXene content.^[^ ^79^ ^]^ Through the wet spinning approach, long fibers are formed by extruding the mixed spinnable solution from the injector and dipping the extrudate in a coagulation bath. Typically, the relatively low aspect ratio and high bending rigidity of MXene flakes have made it difficult to form liquid crystals and be wet spun into continuous fibers alone, so other conductive components and polymer additives, such as GO, PEDOT:PSS, and PU, are usually doped into the solutions to guarantee their spinnability and to perform as flexibility‐improving binders.^[^ ^80^, ^81^, ^82^ ^]^ For example, MXene/PU wet‐spun fibers could be regulated to show either a mixed structure or a sheath/core structure by adjusting the injector, balancing their mechanical properties and energy storage characteristics.^[^ ^81^ ^]^ Recently, a technique for in situ growth of protective layers and ultracompact MXene fibers was reported.^[^ ^83^ ^]^ By combining wet spinning and thermal drawing, a synergy of the cross‐linking reaction (between the MXene and binders) and the stress generated during the thermal drawing process emerged to further enhance the compact alignment of MXene flakes inside the protector. This process unveiled the maximum electrical properties of MXene fibers while maintaining full mechanical performance, supplying a novel strategy for wearable energy storage electronics with demand for resistance against wear and multiple washes. Particularly, Zhang et al. creatively proposed the procedures for wet‐spinning liquid crystalline pure MXene without additives and investigated the generation of different microstructures (opened structure and packed structure) in different coagulation baths (Figure 2d).^[^ ^62^ ^]^ The additive‐free MXene fibers broke through the limitation of electrical conductivity in reported fibers while maintaining flexibility, illustrating the potential to increase energy storage.

Differently, electrospinning can realize a one‐step preparation of nonwoven MXene fabrics by applying a high voltage between the top of the injector and the substrate. The spun fibers exhibit disordered bridging with each other, providing the nonwoven fabrics with flexibility and deformability. The diameter and the cross‐linking morphology of fibers are directly influenced by the concentration of MXenes, the type of solvents, the distance between injector and substrate, and the electric field intensity (Figure 2e). Electrospinning with a moderately volatile solvent usually results in net‐like fabrics with welded bridging points which illustrated excellent mechanical strength.^[^ ^84^ ^]^ Moreover, MXene‐based disordered fibers could be self‐wound on the surface of PET yarns, which provided a new pattern for the fabrication of flexible SCs.^[^ ^68^ ^]^ Taking advantage of the combination of electrospinning and wet‐spinning, Levitt et al. employed an aqueous MXene solution as both a current collector and a coagulation bath to solidify spun PU fibers and attach MXene (Figure 2f).^[^ ^64^ ^]^ The MXene nanoflakes were trapped and embedded into PU fibers, maximizing their interconnection and contact area. The resulting composite fibers provided excellent flexibility, open combination sites, and fast charge transport tunnels, and it demonstrated a promising direction for future fibrous electronics.

#### 2.2.4 Biscrolling Method

**Figure 3:**
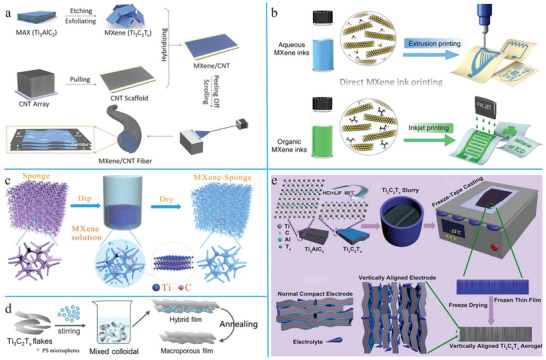
Strategies for the fabrication of MXene‐based flexible materials. a) Flowchart of MXene/CNT fibers by biscrolling with the CNT host. Reproduced with permission.^[^ ^34^ ^]^ Copyright 2018, Wiley‐VCH. b) Schematic illustration of the extrusion printing and inkjet printing for aqueous MXene inks and organic MXene inks, respectively. Reproduced with permission.^[^ ^32^ ^]^ Copyright 2019, Nature Publishing Group. c) The preparation of 3D MXene‐based porous structures with sponge templates. Reproduced with permission.^[^ ^77^ ^]^ Copyright 2020, Elsevier. d) Diagram of the preparation process for macroporous MXene films with sacrificial PS microspheres. Reproduced with permission.^[^ ^85^ ^]^ Copyright 2018, Elsevier. e) The fabrication method for vertically aligned MXene electrodes with ice templates and the structural comparison with the normal compact electrode. Reproduced with permission.^[^ ^69^ ^]^ Copyright 2020, Zhenzhou University.

Biscrolling is a two‐step approach to fabricate composite fibers, by which functional materials are first attached onto host galleries and then the composites are twisted to form helical structures.^[^ ^86^ ^]^ Regarding MXenes, carbon nanotube (CNT) films are introduced as supporting materials to provide extra bonds and flexibility (**Figure** **3**a).^[^ ^34^ ^]^ Based on this, Wang et al. drop‐casted a large amount of MXene dispersion onto the CNT forest and biscrolled the dried MXene/CNT composite film into MXene‐based yarns.^[^ ^31^ ^]^ The prepared yarns with a high MXene loading content up to 98% not only demonstrated superior electrical performance close to that of pure MXene, but also possessed deformability far beyond pure MXene materials, which made them applicable for supercapacitors and batteries.

#### 2.2.5 Printing Methods

Printing is a universal technique for preparing large‐scale, multifunctional, and patterned MXene‐based flexible films. The quality of a printed film is largely dependent on the viscosity of the ink and the surface properties of the substrate used.^[^ ^29^ ^]^ Inks with a high viscosity lead to thick printed layers and increase the likelihood of nozzle blockages, while inks with too low viscosity will be overly fluid and unmanageable. Wen et al. utilized a mixed ink of MXene and *N*‐methyl‐2‐pyrrolidone solvent to diminish the coffee ring effect, a phenomenon of uneven deposition, to some extent, and they made the first attempt to prepare transparent MXene films with inkjet printing.^[^ ^87^ ^]^ This report supplied a method for effective, industrial‐scale preparation of MXene‐based energy storage devices. Due to polarity and volatility differences, aqueous and organic MXene inks without surfactants or additives were separately applied for extrusion printing and inkjet printing, respectively (Figure 3b).^[^ ^32^ ^]^ On the other hand, multipurpose MXene‐based inks with different components and contents have also been used for screen‐printing, fashioning MXene‐based micro‐supercapacitors, lithium‐ion microbatteries (LIMBs), and solar cells. These varied uses illustrate the versatile manufacturing capability of printing techniques.^[^ ^88^ ^]^ Similarly, ink stamping via 3D‐printed cylindrical stamps was also reported to fabricate planar MXene‐based supercapacitors with variable patterns, which expressed high efficiency and convenience.^[^ ^89^ ^]^

#### 2.2.6 Template Methods

Generally, template methods are combined with other MXene fabrication processes to supply special morphologies, such as foamed structures, porous structures, and oriented structures, and provide additional deformability or extra energy storage ability for MXene‐based flexible materials.^[^ ^69^, ^77^ ^]^ For example, applying commercial 3D foam templates joined with a dip‐coating method could fabricate MXene foams with ease, possessing exceptional compression properties and a high occurrence of surface active sites (Figure 3c).^[^ ^77^ ^]^ The mixture of MXene dispersions and sacrificial materials like polystyrene (PS) microspheres were filtered and annealed to remove sacrificial templates and obtain flexible porous structures, which also benefited from electrolyte penetration and efficient electron transport (Figure 3d).^[^ ^85^ ^]^ Similarly, the sacrificial template method was combined with electrospinning to generate nonwoven fabrics with inherently porous structures, which contributed to extra power storage sites and a large buffering capacity.^[^ ^90^ ^]^ With blade‐casting and directional freeze‐drying of MXene aqueous solutions, ice could also perform as the template to produce well‐aligned flexible MXene aerogels.^[^ ^91^ ^]^ Furthermore, even the fabrication of vertically aligned MXene film was realized by this method (Figure 3e).^[^ ^69^ ^]^ The as‐prepared MXene films illustrated nearly thickness‐independent electrical performances and provided promising applications for flexible and wearable electrochemical energy storage devices.

In summary, the choice of fabrication methods for MXene‐based flexible materials emerges as a pivotal determinant not only influencing the ultimate form of the resulting products but also significantly impacting their electrochemical, mechanical, and cyclic performance within the domain of energy storage applications. To illustrate, biscrolling stands out as a promising technique, demonstrating the capacity to yield highly conductive and capacitive devices while preserving their flexibility and stability, as evidenced in Table 1. Furthermore, it is imperative to consider both the constraints and adaptability inherent to various fabrication methodologies. Methods such as coating, biscrolling, printing, and template‐based approaches necessitate additional substrates or templates, with some template methods demanding elevated‐temperature environments. Meanwhile, both printing and coating techniques exhibit potential in meeting the transparency requirements essential for specific applications.

## 3 Application of MXene‐Based Flexible Materials for Batteries

### 3.1 MXene‐Based Flexible Materials for Lithium‐Ion Batteries (LIBs)

Due to their attractive high specific energy, superior cyclic stability and memory effect‐free property, rechargeable LIBs have occupied a leading position in the market of portable electronics and are considered the most promising candidate for future energy storage applications. MXene‐based materials are widely applied in LIBs because they illustrate excellent mechanical flexibility and high electronic conductivity. By preparing MXene‐based materials with loose and flexible porous structures, their volume changes during repeated lithiation/delithiation cycles are buffered to ensure superior cycling stability. However, the inherent structures, preparation processes, and metal current collectors of conventional LIBs bring about their heavy weight and rigidity. Additionally, the active electrode materials coated on current collectors exfoliate easily, which makes it difficult for conventional LIBs to maintain electrochemical performance while bending and twisting. Therefore, the modern LIB design shows failures after repeated deformations, and instead, MXene‐based flexible electrodes have already been developed for flexible batteries with high energy densities and fast charge–discharge processes, named superbatteries.^[^ ^92^, ^93^ ^]^

**Figure 4:**
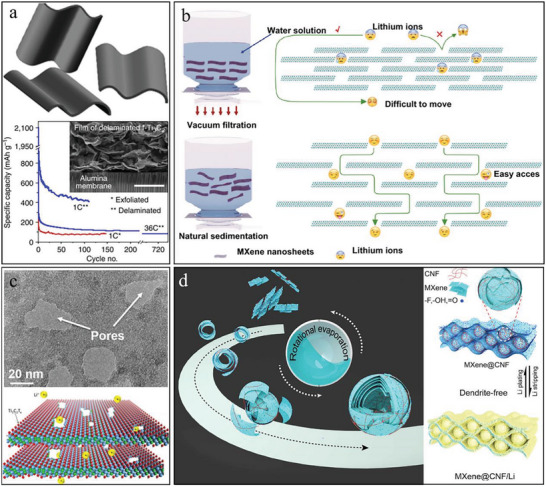
The development of MXene‐based LIBs and methods to avoid the restacking of MXene layers during cyclic process of LIBs. a) The first preparation of stable MXene papers for LIBs and the curves of electrochemical performances. Reproduced with permission.^[^ ^94^ ^]^ Copyright 2013, Nature Publishing Group. b) The schematic comparison of vacuum filtration and natural sedimentation process as well as their Li‐ion transfer ability. Reproduced with permission.^[^ ^95^ ^]^ Copyright 2020, Springer. c) The SEM image and the schematic illustration of the porous MXene/CNT film by chemical etching. Reproduced with permission.^[^ ^96^ ^]^ Copyright 2016, Wiley‐VCH. d) Schematic view of the preparation process for the heterostructured MXene@CNF microspheres and the interlocked structure of the MXene@CNF/Li anode. Reproduced with permission.^[^ ^97^ ^]^ Copyright 2018, Elsevier.

Naguib et al.^[^ ^37^ ^]^ first investigated the possibility of exploiting Ti_3_C_2_ materials as anodes for LIBs. Density functional theory (DFT) calculations illustrated that the possible Li‐storage mechanism was Li ions intercalating into the vacant spaces between Ti_3_C_2_ layers, and the theoretical capacity of the structure was 320 mA h g^−1^. Later, an MXene paper with a stable structure was first prepared by the filtration of MXene dispersion (**Figure** **4**a).^[^ ^94^ ^]^ The flexible paper illustrated a capacity of 410 mA h g^−1^ at a cycling rate of 1 C and retained 110 mA h g^−1^ at 36 C, opening the door for the application of MXene‐based flexible materials in battery electrodes. The open structure of flexible MXene papers effectively enhanced their electrochemical performances, but inevitably, interlayer collapse and self‐stacking cause decreases in conductivity and electrolyte accessibility. The dual effects of decreased expansion paths and reduced diffusion kinetics severely impeded the transportation of ions, resulting in the low reversible capacity of only 100–200 mA h g^−1^ for the original MXene films, which was far below the theoretical value.^[^ ^94^, ^96^, ^98^ ^]^

In order to solve this problem, scholars have conducted many studies on avoiding the restacking of MXene layers, facilitating fast ion transport and thus realizing rapid charge–recharge processes. Vacuum filtration was the most commonly used approach for preparing MXene papers, but this process also caused overly compact stacking and small interlayer distances.^[^ ^95^ ^]^ Compared with the thickness of vacuum‐filtered MXene films (3.31 µm), the naturally deposited ones of the same weight possessed the thickness of 4.05 µm (22% increased), which illustrated the obviously enlarged interlayered distances (Figure 4b). With this technique, the diffusion barrier of ions was lowered and the ion accessibility between MXene layers was improved, providing higher capacity and better rate performance. The reversible capacity of naturally deposited MXene films was nearly twice that of vacuum‐filtered ones and reached a capacity of 351 mA h g^−1^ at 30 mA g^−1^, a largely enhanced rate and cyclic stability approaching the theoretical value of Ti_3_C_2_T*_x_* MXenes. The porous Ti_3_C_2_T*_x_* (p‐Ti_3_C_2_T*_x_*) obtained by chemical etching could also be utilized for the preparation of flexible and hetero MXene/CNT films (Figure 4c).^[^ ^96^ ^]^ Compared with original Ti_3_C_2_T*_x_*‐based films, the as‐prepared p‐Ti_3_C_2_T*_x_*/CNT films illustrated a significantly improved Li storage capacity of 1250 mA h g^−1^ at 0.1 C, tremendous cyclic stability, and a favorable rate property (330 mA h g^−1^ at 10 C).^[^ ^99^ ^]^ In order to further resolve the problems of limited storage space and low ion diffusion rate in the vertical direction caused by layer‐by‐layer restacking, Wang et al. proposed a new strategy for the large‐scale preparation of freestanding, light‐weight, ultrathin (≈25 µm), and flexible MXene papers by spin‐steaming a mixed colloid solution of MXene nanoflakes and cellulose nanofibers (CNFs).^[^ ^100^ ^]^ MXene and CNF first formed layered micelles by intermolecular hydrogen bonding, which gradually curled to form spherical micelles as a result of the drum granulation effect (Figure 4d).^[^ ^97^ ^]^ MXene@CNF microspheres produced by the LbL self‐assembly method were finally combined with layered micelles to generate a sandwiched structure. The layered materials and microspheres produced interlocked interfaces to tremendously enhance toughness in the vertical direction, to maintain the lateral flexibility of the MXene film, and to prevent the restacking of MXene layers. The as‐prepared composite papers illustrated outstanding mechanical flexibility to be easily bent or folded into various shapes without obvious damage. The obtained MXene@CNF/Li anode could perfectly match with a freestanding LiFePO_4_/CNF (LFP@CNF) cathode to construct a completely flexible lithium metal battery with a high specific capacity and outstanding stability, which exhibited an excellent capacity retention of over 95.25% during ≈100 cycles and demonstrated superior long‐term durability.

**Figure 5:**
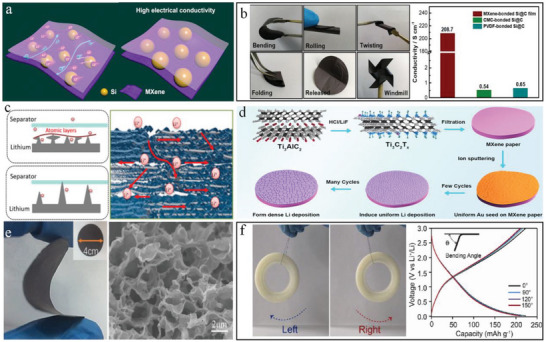
Different strategies for the enhancement of MXene performances in LIBs. a) Schematic illustration of the synergy of Si and MXene with high electrical conductivity. Reproduced with permission.^[^ ^26^ ^]^ Copyright 2019, American Chemical Society. b) Images of the MXene‐bonded Si@C film with several deformations and the conductivity comparison of MXene‐bonded Si@C, CMC‐bonded Si@C, and PVDF‐bonded Si@C. Reproduced with permission.^[^ ^101^ ^]^ Copyright 2019, Wiley‐VCH. c) Illustration of a roll‐to‐roll prepared MXene/Li film and the constraint of Li dendrites growth. Reproduced with permission.^[^ ^102^ ^]^ Copyright 2017, Elsevier. d) The process of Li growth on Au‐coated MXene. Reproduced with permission.^[^ ^103^ ^]^ Copyright 2021, Elsevier. e) Digital photograph and SEM image of the flexible 3D porous MXene film. Reproduced with permission.^[^ ^104^ ^]^ Copyright 2019, Wiley‐VCH. f) The tungstate/MXene fiber to hang a 10 g tape with repeated swinging and the voltage–capacity curves at different bending angles. Reproduced with permission.^[^ ^105^ ^]^ Copyright 2020, Elsevier.

One of the most effective methods to reach the full potential of flexible MXenes in high‐performance LIBs is to synergistically combine them with other anode materials.^[^ ^106^, ^107^ ^]^ Silicon (Si) was one of the most ideal anode materials for LIBs due to its highest specific capacity (3579 mA h g^−1^ at moderate temperature), low working potential, and natural abundance.^[^ ^108^, ^109^ ^]^ However, the large volume expansion during operation and poor conductivity impede its practical applications.^[^ ^110^, ^111^ ^]^ The synergy of Si and flexible MXenes show promise to illustrate an unexpected effect, due to the relatively high conductivity and outstanding mechanical performances of the latter (**Figure** **5**a).^[^ ^26^ ^]^ The flexible, independent, and binder‐free Si/MXene composite papers, prepared by vacuum‐filtering the mixed dispersion of silicone nanospheres and MXene flakes, could be directly utilized as the anode of LIBs. MXene flakes were utilized as 2D supports onto which Si nanospheres were loaded, preventing the aggregation of Si particles and the associated strain induced during the lithiation/delithation process. Moreover, the conductive MXene network provided a rigid collector that enhanced the conductivity of the composite film, adapted to large volume expansion, supplied additional active sites, and facilitated effective ion transport. Simultaneously, Si nanospheres separated MXene nanoflakes to avoid restacking and the resulting independent architecture contributed to its tremendous performance, with an excellent capacity of 2118 mA h g^−1^ at 200 mA g^−1^ after 100 charge/discharge cycles. Wrapping the surfaces of Si nanoparticles with a carbon layer could further enhance the conductivity of composite Si@C particles and suppress the volume change of the Si anode.^[^ ^101^ ^]^ Through vacuum filtration, exfoliation, and drying, the MXene‐bonded Si@C film was facilely fabricated and could withstand various mechanical deformations such as bending, twisting, rolling, folding, and even being cut and folded to a windmill. Besides significantly enhancing the conductivity, the 3D porous structure of MXene‐bonded Si@C film supplied more spaces for buffering the great volume expansion/contraction of Si@C nanoparticles and facilitated electrolyte penetration, which raised the durability and charge transmission process of the electrode (Figure 5b). An et al. drew a similar conclusion that LIBs with the MXene/layer‐by‐layer‐assembled Si/C (MXene/L‐Si/C) host illustrated an ultralong durability of 500 h in a carbonate‐based electrolyte.^[^ ^112^ ^]^ In addition to Si nanoparticles, transition metal oxides have been synergized with MXenes due to their high theoretical capacity, moderate cost, and environmental friendliness. For example, Ahmed et al. applied an atomic layer deposition technique to generate a flexible SnO_2_/MXene anode for LIBs, and they further improved its performance by depositing a thin HfO_2_ passivation layer onto it.^[^ ^113^ ^]^ The as‐prepared electrode retained a capacity of 843 mA h g^−1^ after 50 cycles.

Although MXenes have been considered as the most attractive and prospective anode for next‐generation LIBs, their lithophobicity impedes the deposition of Li. To effectively solve this problem, Li et al. utilized a roll‐to‐roll method to fabricate a flexible layered Ti_3_C_2_/Li film based on the ductility of lithium and the lubricity of the atomic layers.^[^ ^102^ ^]^ Through this approach, the lithophobicity of MXene effectively prevented the nucleation and growth of lithium dendritic crystals and constrained the occurrence of electroplated lithium in the nanoscale gaps of Ti_3_C_2_ MXene layers (Figure 5c). The high conductivity and surface nucleation sites of MXene nanoflakes benefited both electron and lithium ion transport, resulting in a flat potential curve with only 1.5% increase of the lowest overpotential during 200 cycles. The introduction of a lithophilic metal layer on the surface of MXenes could also lower the nucleation barrier of lithium, inducing uniform lithium deposition and preventing the growth of harmful lithium dendritic crystals.^[^ ^114^ ^]^ For example, an Au coating prompted the flat and dense deposition of lithium due to its ability to spontaneously alloy with Au (Figure 5d).^[^ ^103^ ^]^ Compared to the high Li nucleation barrier of MXene paper (42.7 mV), the Au thin film illustrated a barrier of nearly zero (0.3 mA h cm^−2^ at 0.1 mA cm^−2^). Similarly, indium (In) could also be applied to decorate the flexible MXene paper and increase its lithophilicity.^[^ ^115^ ^]^ The Li*_x_* In*_y_* layer was generated on the In‐MXene paper by an alloy reaction with almost undetectable Li nucleation overpotential. Other materials like covalent organic frameworks and Co‐NiS were have been introduced for the preparation of MXene‐based flexible films, which decreased Li nucleation barrier and accomplished dense Li deposition without dendritic crystals.^[^ ^116^, ^117^, ^118^ ^]^

In addition to vacuum filtration, other approaches were reported to fabricate flexible MXenes that could withstand strain. Zhao et al. prepared the MXene/S film and sublimated S particles in an Ar atmosphere at 300 °C for 3 h to obtain an MXene foam with 3D porous architecture and toughness (Figure 5e).^[^ ^104^ ^]^ Due to the dense accumulation of layered MXene nanosheets, the compact MXene film exhibited an ultralow specific area of only 1.9 m^2^ g^−1^ and a pore volume of only 0.03 cm^3^ g^−1^ with Brunauer–Emmett–Teller (BET) analysis. By contrast, the BET pore volume of the microporous MXene foam could reach as high as 4.34 cm^3^ g^−1^, which could provide abundant active sites for lithium storage, facilitate electrolyte penetration, and fasten Li^+^ transport. The porous MXene foam manifested a superior capacity of 455.5 mA h g^−1^ at a current density of 50 mA g^−1^, an outstanding rate performance with 101 mA h g^−1^ capacity retention even at 18 A g^−1^, and a long‐term durability of 3500 cycles. Moreover, the 3D MXene‐based composite foam provided an efficient solution for the large volume changes of Li‐ion batteries brought by other active anode materials, such as Co_3_O_4_.^[^ ^119^ ^]^

In recent years, fibrous batteries have become more popular as the ideal energy supply for wearable electronics. Wang et al. continuously injected a mixed liquid crystal (LC) colloid into a positively charged coagulation bath with chitosan cross‐linker and produced spun tungstate/MXene fibers through electrostatic interaction.^[^ ^105^ ^]^ After that, the fibers were dipped into hydroiodic acid to etch the protonated chitosan molecules between MXene nanoflakes, eventually acquiring freestanding and highly aligned tungstate/MXene fibers. The as‐prepared fibers demonstrated an ultrahigh tensile strength of ≈220 MPa, and a single tungstate/MXene fiber could maintain completeness while withstanding a 10 g tape and suffering from repeated rising and swinging processes (Figure 5f). Besides superior mechanical performance, this fibrous LIB reached a high reversible capacity of 135.3 mA h g^−1^ at a large current of 0.428 mA and a high retention of 126.0 mA h g^−1^ even after 2000 cycles, demonstrating its prominent electrochemical characteristics. The Li ion storage property of flexible composite fiber under mechanical deformations was also investigated in detail. Compared with the initially undeformed state, no obvious changes were observed on the charge/discharge curves of the fibers with the various bending angles of 90°, 120°, and 150°, respectively. A high capacity retention of 93% was shown at a large bending angle of 150° and that of over 75.95% was achieved during 1000 bending cycles at 90°.

### 3.2 MXene‐Based Flexible Materials for Lithium–Sulfur Batteries (LSBs)

Compared with LIBs (with the an energy density of ≈300 W h kg^−1^), LSBs that can provide a higher energy density of 2600 W h kg^−1^ and have attracted more and more recent attentions.^[^ ^120^, ^121^, ^122^ ^]^ However, the large volume expansion, poor electrical conductivity, and severe shuttle effect of LSBs prevent them from further development and widespread applications.^[^ ^123^, ^124^ ^]^ Typically, the shuttle effect refers to the disassociation of long chain lithium polysulfides (Li_2_S*_n_*, 4 ≤ *n* ≤ 8, known as LiPSs) generated during discharge process and their shuttle between two electrodes.^[^ ^125^, ^126^ ^]^ This negative effect will cause the loss of active materials and results in the fast attenuation of capacity, poor cycling performance, and the low Coulombic efficiency. Moreover, LiPSs will react with the lithium anode to form an insulation layer which results in significant overpotential. Simultaneously, the isolating nature of S tremendously weakens the redox reaction kinetics and thus decreases the rate performance of devices and the efficient utilization ratio of S.^[^ ^127^ ^]^ 2D MXenes, especially Ti*_x_* C*_y_* groups, show strong chemical affinity to LiPSs and significantly diminish their migration. Furthermore, the open structure of MXenes can effectively suppress the severe volume expansion of LSBs and further enhance their capabilities.

**Figure 6:**
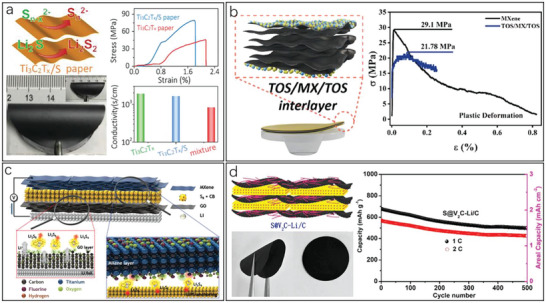
Flexible MXene‐based functional layers and the corresponding property enhancement strategies for LSBs. a) Scheme and digital photograph of the flexible Ti_3_C_2_T*_x_*/S paper and the curves of property comparison. Reproduced with permission.^[^ ^128^ ^]^ Copyright 2019, Wiley‐VCH. b) Illustration of TOS/MX/TOS film and the stress–strain curves comparing TOS/MX/TOS with MXene. Reproduced with permission.^[^ ^129^ ^]^ Copyright 2022, Elsevier. c) Schematic diagram of a flexible film composed of GO, S, and Ti_3_C_2_T*_x_* MXene (GSM) with the current collector of MXene and the selective separator of GO. Reproduced with permission.^[^ ^130^ ^]^ Copyright 2021, Wiley‐VCH. d) Illustration, photograph and curves of a S@V_2_C‐Li/C electrode with expanded interlayer spaces, high conductivity, and large capacity. Reproduced with permission.^[^ ^131^ ^]^ Copyright 2020, American Chemical Society.

In 2015, Nazar's group first reported the application of MXene in LSBs, which illustrated an outstanding cyclic stability and a high capacity up to 70 wt% S.^[^ ^132^ ^]^ X‐ray photoelectron spectroscopy further demonstrated that the strong interaction of polysulfides with surface Ti atoms and hydroxy groups could enhance the chemical absorption of LiPSs, diminishing their shuttle and eventually obtaining LSBs with high performances. The flexible Ti_3_C_2_T*_x_*/S paper could be prepared by combining Ti_3_C_2_ MXene with S through physical vapor deposition (PVD).^[^ ^128^ ^]^ Since the doping of S did little harm to the intrinsic toughness of MXene film, the freestanding composite paper could maintain intactness with convex and concave bending to a large extent and demonstrated a similar mechanical strength and a slightly decreased fracture strain (from 2.2% to 1.8%) compared with that of the pure Ti_3_C_2_T*_x_* paper (**Figure** **6**a). The prominent flexibility of Ti_3_C_2_T*_x_*/S electrode is attributed to the ultrahigh mechanical strength of lamellar MXene nanoflakes as well as their bridged network generated by capillary force during the vacuum filtration process. After bending and releasing in different ratios and 25 bending cycles, the resistances of Ti_3_C_2_T*_x_*/S papers remained almost unchanged, demonstrating that the interlinked nanoflake network could absorb the induced stress and withstand the cyclic stress while maintaining intactness. The outstanding mechanical strength of the composite electrodes not only ensured their fast electron/ion transport kinetics under deformation, but also imbued them with the capacity to withstand the volume expansion of S. Further, the polysulfides and the polar MXene mediators had a unique reaction that could grow a thick sulfate complex layer in situ, which performed as a protector to suppress the shuttle effect of LiPSs and enhance the utilization ratio of S. A flexible LSB was further generated by subsequently assembling the Ti_3_C_2_T*_x_*/S paper, separator, and lithium foil, and an ultrahigh capacity of 1119 mA h g^−1^ was reached even under a bending state because of the compact structure and the effective charge transport.

Doping other active materials into MXene anodes was reported as an effective method to further explore shuttle suppression capability. Yao et al. introduced a TiS_2_/TiO_2_ heterostructure into the freestanding, sandwiched film, known as TOS/MX/TOS interlayer, by vulcanizing the vacuum‐filtered s‐Ti_3_C_2_T*_x_* thin film through a CVD process (Figure 6b).^[^ ^129^ ^]^ The TOS/MX/TOS film with high‐temperature treatment still demonstrated superior toughness and prominent mechanical performance due to the interlinked 3D structure of s‐Ti_3_C_2_T*_x_* nanosheets. The flexible composite film expressed no failures even after twice completely folding and expanding, and its stress–strain curve indicated the largest tensile stress and the largest elastic deformation of 30 MPa and 0.82%, respectively. Similarly, the various oxygen‐containing groups of GO could also suppress the loss of S and fix LiPSs, and a flexible film composed of GO, S, and Ti_3_C_2_T*_x_* MXene was directly produced by vacuum filtration.^[^ ^130^ ^]^ The MXene side with high conductivity and excellent mechanical properties was utilized as the current collector, while the insulated and highly porous GO layer performed as the selective separator (Figure 6c). The prepared flexible LSB could light 50 LEDs in a bending state, which exhibited its application potential in wearable electronics. Taking advantage of the metastable S_2–4_ molecules and excluding S_5–8_ with larger size from ultra‐microporous carbon (UMC) matrix was another approach to intrinsically restrain the shuttle effect of LiPSs.^[^ ^133^ ^]^ The MXene was introduced into the DMF dispersion of S_2–4_/UMC composite as a binder and a MXene‐based flexible film was generated by vacuum filtering, which constructed a tough and continuous conductive network to enhance the mechanical and electrochemical performance of the electrode.^[^ ^134^ ^]^ The flexible MXene‐bonded S_2–4_/UMC electrode illustrated a superior capacity of 1029.7 mA h g^−1^ at 0.1 C and indicated a high cyclic stability by maintaining 946.7 mA h g^−1^ after 200 charge/discharge cycles.

Another key factor for achieving high performance of LSBs at high current density is the Li‐ion transport ability. Typically, a successive Li‐ion transporting path is unable to be generated in S electrodes regardless of the complexity of S nanostructures, which results in the poor electrochemical performance of LSBs (especially with high S content or at high current density). Therefore, a method to provide continuous Li‐ion access in S‐containing electrodes is effective at improving the entire electrochemical performance of LSBs. Chen et al. prepared a S@V_2_C‐Li composite with high Li‐ion conductivity, in which the unique nanostructured V_2_C‐Li MXene was applied as the host for loading sulfur in order to raise the rate property of LSBs.^[^ ^131^ ^]^ In detail, the Li ions were intercalated into the interlayer of V_2_C MXene to expand its lamellar distance, which lowered the energy barrier of ion exchange and migration and provided continuous interior tunnels for Li‐ion transport. This special characteristic along with the strong polysulfide affinity of the prepared S@V_2_C‐Li/C electrode enabled its small capacity attenuation of 0.053% and 0.051% after 500 cycles at high current densities of 1 C and 2 C, respectively (Figure 6d).

Besides MXene papers, fiber‐shaped flexible MXene electrodes have also shown promise in LSBs. Liu et al. reported a MXene‐based flexible fibrous electrode by subsequently drop‐coating pure Ti_3_C_2_T*_x_* nanosheets and Ti_3_C_2_T*_x_* nanosheets with in situ loaded S (Ti_3_C_2_T*_x_*/S) onto a common fabric.^[^ ^135^ ^]^ The terminal groups of Ti_3_C_2_T*_x_* (─OH and ─O) were bonded with the fabric ─OH groups through van der Waals force, and the conductive frame of MXene‐coated textile fabric also compactly integrated with Ti_3_C_2_T*_x_*/S to form an LbL structure. This structure produced an effective physical encapsulation that not only prevented the nanosheets from exfoliation, but also accommodated the volume change during charge/discharge process, which suppressed the loss of electroactive S and maintained structure integrity. Meanwhile, the heterostructure provided a strong chemical absorption for LiPSs and improved the utilization of S during cycling. Therefore, the MXene‐based flexible electrode demonstrated a high initial capacity (916 mA h g^−1^ at 1 C) and an ultralong cyclic stability (674 mA h g^−1^ after 1000 cycles at 1 C). More importantly, the flexible integrated LSB could lighten LED lights at different bending angles of 0°, 90°, 180°, 360°, and 720° without obvious change of luminance. Li et al. prepared a fibrous rGO/MXene@S anode through wet spinning technique, in which sulfur was uniformly distributed and compactly enveloped between graphene and MXene nanosheets.^[^ ^136^ ^]^ The assembled electrode illustrated a high discharge capacity of 1483.1 mA h g^−1^ at 0.1 C, an excellent rate performance of 733.3 mA h g^−1^ at 2 C and a superior long‐term stability of only 0.043% per cycle attenuation at 1 C for over 1000 cycles.

In addition to applying MXene‐based materials as electrodes, the introduction of flexible MXene/polymer separators is also considered as an effective strategy to impede the migration of dissociated LiPSs between two electrodes. By vacuum filtering the 2D delaminated Ti_3_C_2_ (d‐Ti_3_C_2_) nanoflakes, Dong et al. introduced a MXene layer onto a polypropylene (PP) separator to prepare the d‐Ti_3_C_2_/PP composite separator.^[^ ^137^ ^]^ After that, the flexible all‐MXene electrode was fabricated by coating the slurry containing S‐loaded 3D alkalized Ti_3_C_2_ MXene nanoribbon on the surface of the as‐prepared d‐Ti_3_C_2_/PP film. The integrated electrode performed at a high and reversible electrical capacity of 1062 mA h g^−1^ at 0.2 C as well as a significant rate capacity of 288 mA h g^−1^ at 10 C. Moreover, other materials like commercial Celgard separator and glass fiber have all been reported composites with MXenes to enhance electrical conductivity as well as trap polysulfides, eventually accomplishing LSBs with favorable electrochemical performances.^[^ ^138^, ^139^ ^]^

### 3.3 MXene‐Based Flexible Materials for Other Batteries

With the widespread utilization of LIBs and the continuous consumption of lithium resources, the development of non‐Li‐ion batteries (NLIBs) becomes more and more urgent.^[^ ^140^ ^]^ In recent years, many NLIBs perform as fascinating candidates due to their superior properties and emerging low‐cost techniques. Among them, 2D MXene layers intercalated with alkali‐metal ions like Na, K, other potential metal ions like Mg, Al, and Zn, and aqueous protons are simulated and experimentally verified to possess relatively high energy storage capacities.^[^ ^141^, ^142^, ^143^, ^144^, ^145^, ^146^ ^]^ In this section, we will introduce the preparation of flexible anodes for rechargeable batteries by combining MXenes with Na, K or Zn elements.

#### 3.3.1 Sodium‐Ion Batteries (SIBs)

**Figure 7:**
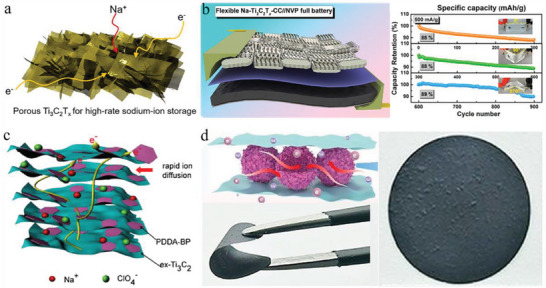
Flexible MXene‐based electrodes for SIBs and the related performance improvement methods. a) Scheme of the porous Ti_3_C_2_T*_x_* for facilitated sodium‐ion storage. Reproduced with permission.^[^ ^147^ ^]^ Copyright 2018, American Chemical Society. b) Illustration of the flexible Na–Ti_3_C_2_T*_x_*–CC/NVP SIBs and the capacity retention at different bending angles. Reproduced with permission.^[^ ^148^ ^]^ Copyright 2020, American Chemical Society. c) Diagram of the heterostructured PDDA–BP/Ti_3_C_2_T*_x_* electrode for SIBs with effective ion diffusion path. Reproduced with permission.^[^ ^149^ ^]^ Copyright 2019, Elsevier. d) The composite MXene film for SIBs with active MoS_2_‐contained 3D hollow spheres and the flexibility of bending to 180°. Reproduced with permission.^[^ ^150^ ^]^ Copyright 2022, Elsevier.

Rechargeable SIBs have become an intriguing alternative of LIBs due to the similar low potential of Na and Li (−2.71 V vs SHE and −3.04 V vs SHE, respectively), their cost‐efficient price, and the abundant resources of Na. However, the radius of Na atom (1.06 Å) is larger than that of Li atom (0.76 Å), which causes slower sodiation/desodiation kinetics and more severe volume expansion. MXenes with high conductivity, large interlayer distance and low Na^+^ diffusion barrier may facilitate the fast diffusion of Na ions (**Figure** **7**a), and thus enable their use in flexible SIBs.^[^ ^147^, ^151^ ^]^ However, predictions also indicate that the larger relative radius of sodium cations lead to a lower theoretical capacity of SIBs (315.8 mA h g^−1^) than that of LIBs.^[^ ^142^ ^]^ Therefore, it has been of urgent importance to design novel MXene‐based flexible materials with proper interlayers and suitable structures for easy sodiation/desodiation processes.

Similar to other 2D materials, MXene nanoflakes show a tendency for self‐stacking, which limits their performance in flexible energy storage electronics. In order to fully exploit the electrochemical energy storage capability of MXenes, strategies such as removal of loaded sulfur on Ti_3_C_2_T*_x_* nanosheets to increase porosity and acid‐induced crumpling were developed to improve the storage kinetics of sodium ions and to enhance the battery properties.^[^ ^147^, ^152^ ^]^ However, these MXene films revealed relatively poor mechanical performances, and alternative preparations such as attaching MXenes on common flexible substrates were explored. For example, the flexible Na–Ti_3_C_2_T*_x_*–CC electrode was obtained by depositing MXene on flexible carbon clothes (CC) and infusing molten metallic Na.^[^ ^153^ ^]^ The Ti_3_C_2_T*_x_* could effectively induce the nucleation and lateral‐oriented deposition of Na, which avoided the generation of moss‐ and branch‐like crystals and achieved a smooth and schistose morphology of Na surface inherited from the atomic alignment of MXene (Figure 7b). This Na–Ti_3_C_2_T*_x_*–CC paper could maintain intactness with repeated folding and rolling tests, illustrating its promising applications in wearable, deformable, and shaped batteries. Gogotsi's group took advantage of the strong interactions between MXene nanoflakes and polymethyl methacrylate (PMMA) microspheres to fabricate MXene films with hollow structure, utilizing annealing treatment as the PMMA template removal method.^[^ ^154^ ^]^ The 3D MXene films with large pores were freestanding and flexible enough to exhibit superior mechanical properties, and the favorable contact between microspheres along with the conductive properties of MXenes provided the resulting film with favorable conductivity. Additionally, the porous films could be directly applied as anodes for Na^+^ storage without extra collectors or binders. With a low charge rate of 0.25 C, 3D Ti_3_C_2_T*_x_*, V_2_CT*_x_*, and Mo_2_CT*_x_* electrodes reached reversible capacities of ≈330, 340, and 370 mA h g^−1^, respectively, and also held remarkable rate ability as well as long‐term durability.

Doping other electrochemically active nanomaterials into MXenes has been found to not only prevent Ti_3_C_2_T*_x_* from restacking, but also benefit their Na storage capability. 1D CNT materials were doped into the lamellar structure of 2D MXenes, and the 2D/1D hybridization effectively diminished the stacking between nanosheets as well as produced lamellar MXene‐based films with a fluffy structure. The flexible MXene/CNT film could directly perform as the freestanding electrode for Na^+^ storage, which realized a prominent volumetric capacity of 421 mA h cm^−3^ at 20 mA g^−1^ and still retained a capacity as high as 89 mA h cm^−3^ at a current density of 5000 mA g^−1^. BP was one of the most promising materials to combine with MXenes and prepare flexible anodes for SIBs.^[^ ^155^ ^]^ The surface‐to‐surface parallel contact between two 2D materials generated a molecular‐level heterostructure and provided effective charge transfer and diffusion tunnels. Additionally, the heterostructure indicated a potential to buffer the severe volume change and to prevent the congregation and loss of BP (induced by the phase transition and structure collapse during rapid charge/discharge processes), which was crucial for further improving the reversible capacity and the long‐term cycling durability.^[^ ^156^ ^]^ Similarly, Zhao et al. reported a noteworthy electrostatic attraction method to fabricate flexible anodes based on molecular leveled poly(diallyldimethylammonium chloride) (PDDA)–BP/Ti_3_C_2_ heterostructures (Figure 7c).^[^ ^149^ ^]^ This electrode exhibited a reversible capacity of 1112 mA h g^−1^ at 0.1 A g^−1^ and demonstrated a superior cyclic stability at 1.0 A g^−1^. By intercalating the carbonyl‐based organic salt‐disodium rhodizonate (Na_2_C_6_O_6_) (which possesses high gravimetric capacity) into the 2D MXene layers, a flexible, binder‐free organic cathode was prepared for highly stable and fast Na storage.^[^ ^157^ ^]^ Not surprisingly, MXene nanosheets in the composite paper provided a conductive network and sufficient sites for Na‐ion transport and loading, as well as protecting the Na_2_C_6_O_6_ nanoparticles from dissolving in the electrolyte.

Layered transition metal dichalcogenides, such as VSe_2_, WS_2_, VS_2_, and MoS_2_, possess unique layered structures, high theoretical specific capacities, and advantageous physical and chemical performances, allowing them to construct van der Waals heterostructures with distinct electrochemical properties in combination with highly conductive MXenes.^[^ ^158^, ^159^, ^160^ ^]^ For example, the active MoS_2_‐contained 3D hierarchical hollow spheres (H‐MoS2@NC) doped with a Ti_3_C_2_T*_x_* MXene binder was used to construct a MXene‐H‐MoS_2_@NC composite electrode by LbL vacuum filtration.^[^ ^150^ ^]^ The electrode expressed brilliant flexibility, withstanding bending to 180° without fractures and further greatly diminished the volume expansion during charge/discharge process (Figure 7d). Due to the advantageous structures of anode and cathode materials, the assembled half/full SIBs illustrated remarkable sodium storage property, high rate capacity, and long‐term cycling stability. Similarly, Yuan et al. applied a hydrothermal treatment and the electrostatic self‐assembly technique to synthesize a flexible 3D MoS_2_‐based sodium‐ion anode with Nb_2_CT*_x_* MXene bonding and a hollow carbon sphere frame.^[^ ^22^ ^]^ This strategy successfully enhanced the utilization of carbon sphere skeletons and decreased the diffusion distance of Na^+^, which increased the specific capacity and rate property. Moreover, Nb_2_CT*_x_* flakes could efficiently restrain the MoS_2_ exfoliation and reduce capacity attenuation, which ensured the long cycling life. These carbon‐loaded MXene/MoS_2_ anodes retained the initial structure at the bending angles of 90°, 180°, and 210°, demonstrating their outstanding mechanical capabilities. SIBs could further be prepared with these flexible films and were still capable of lighting LED bulbs at a bending angle of 90°.

#### 3.3.2 Potassium‐Ion Batteries (PIBs)

K expresses a lower reduction potential (−2.93 V vs SHE) and a higher theoretical capacity (687 mA h g^−1^) than that of Na, matching closely with Li and therefore making K one of the best choices for LIB alternatives.^[^ ^161^, ^162^ ^]^ However, K ions illustrate an even larger radius than those of Li and Na, which is likely to cause more severe volume variation during potassiation/depotassiation process and thus limits the material choices of electrode for PIBs.^[^ ^163^ ^]^ Early calculations verified the possibility for K ions to embed into 2D conductive MXenes with large interlayer spaces, suggesting MXenes as potential high‐capacity anode materials for PIBs.^[^ ^142^ ^]^ Subsequently, 2D MXene materials involving Ti_3_CNT*_x_*, Ti_3_C_2_T*_x_*, and Nb_2_CT*_x_* were experimentally developed as components of anode materials for PIBs.^[^ ^164^, ^165^ ^]^ Naguib et al. first explored the electrochemical properties of Ti_3_C_2_T*_x_* as electrodes for PIBs, and the resulting curves retained a reversible capacity of 75 mA h g^−1^ after 100 charge/discharge cycles.^[^ ^166^ ^]^ Thus, the ongoing construction of these particular MXene‐based flexible electrodes has been extremely crucial to further improve potassium storage ability and cycling stability during PIB applications.

**Figure 8:**
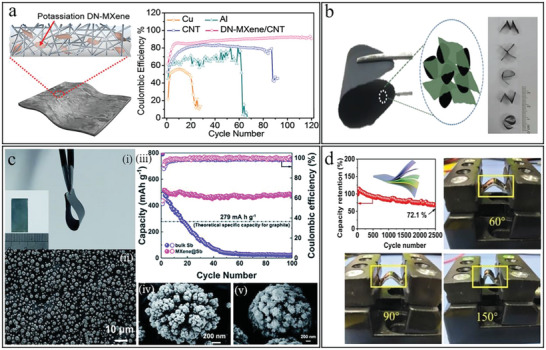
The flexible MXene‐based electrodes for PIBs and ZIBs. a) Scheme of the MXene/CNT film with potassium storage and the Coulombic efficiency of different electrodes for PIBs during charge cycles. Reproduced with permission.^[^ ^167^ ^]^ Copyright 2020, Wiley‐VCH. b) The MXene‐bonded HC flexible film for PIBs and the five cut characters of “MXENE”. Reproduced with permission.^[^ ^25^ ^]^ Copyright 2019, Wiley‐VCH. c) MXene@Sb paper for PIBs. i) Digital photograph of the MXene@Sb composite with a bending angle of 180°. ii, iv) SEM images of the MXene‐based anode with different magnifications. iii) Cyclic capacity performance and Coulombic efficiency of bulk Sb and MXene@Sb. v) SEM image of the MXene‐based anode after 100 cycles. Reproduced with permission.^[^ ^168^ ^]^ Copyright 2019, The Royal Society of Chemistry. d) Cyclic capacity of VO_2_/MXene anode for ZIBs and the VO_2_/MXene flexible film at the bending angles of 60°, 90°, and 150°. Reproduced with permission.^[^ ^169^ ^]^ Copyright 2021, Wiley‐VCH.

Combining other low‐dimensional materials with MXenes can enhance the performance of MXene‐based flexible electrodes in PIBs. A freestanding flexible film produced by the assembly of nitrogen‐containing and defect‐abundant MXene with CNT could be utilized as the storage host for metallic K.^[^ ^167^ ^]^ The highly potassiphillic MXene nanosheets contained in the electrode were available to serve as “seed sites” to initiate K nucleation during circulations, preventing the direct electroplating of K outside the frame and avoiding the formation of dendritic crystals (**Figure** **8**a). Because of this unique property, the prepared flexible electrode possessed a high Coulombic efficiency of 98.6%, as well as low overpotential and a long cycling life during repeated K electroplating/exfoliation procedures. Cao et al. hybridized MXene nanoflakes and graphite nanosheets by ultrasonic treatment in solution to prepare a stable and uniform dispersion, and then generated a freestanding flexible electrode by vacuum‐assisted filtration. The composite electrode indicated a prominent toughness to withstand folding tests without fracturing. Compared with conventional PVDF binders, MXene frames with distinctive flexibility could undergo structural deformation without degradation, which allowed the electrode to remain intact and ensured cyclic stability during potassium storage periods.^[^ ^170^ ^]^

Other anode materials for PIBs have also been reported in combination with MXenes for performance enhancement. Hard carbon (HC) with large layer separations and amorphous structure has been thought suitable for K^+^ storage, but its independent cycling stability has not yet achieved satisfactory levels. Integrating the 3D open‐structured MXene with the HC found remarkable success in accomplishing both high capacity and superior cycling stability.^[^ ^25^ ^]^ High‐magnification SEM images illustrated that HC particles act as spacers to prevent the restacking of MXene flakes, which increased the interlayer distances of MXene from 12.2 Å (of pure MXene film) to ≈14.2 Å (of HC‐MXene film). The MXene‐bonded HC film was freestanding and flexible to be freely folded or bent to any shapes and patterns, such as forming the five characters of “MXene” after a simple cutting process, which illustrated their great potential to perform as electrodes for flexible secondary batteries (Figure 8b). When applied in PIBs, this electrode could provide a high capacity of 210 mA h g^−1^ at 50 mA g^−1^ and a favorable capacity retention of 84% after 100 cycles, which significantly exceeded the performances of conventional PVDF‐bonded HC electrodes (36.8% capacity retention). Additionally, antimony (Sb) could react with K to synthesize K_3_Sb, providing a high theoretical specific capacity (660 mA h g^−1^) with a safe working potential, high conductivity, and large physical density (6.7 g cm^−3^).^[^ ^171^ ^]^ The steady growth of lamellar and porous Sb on the MXene paper could supply short diffusion distances for potassium ions and realize fast and accessible ion transport. The in situ growth of hierarchically porous Sb by electrical deposition was also reported to produce a flexible freestanding MXene@Sb paper without binder.^[^ ^168^ ^]^ The composite paper could be easily bent to ≈180° without any obvious damage to the structure. The MXene@Sb anodes applied for PIBs demonstrated an ultrastrong potassium storage performance. In detail, an excellent reversible capacity retention of 94.32% and a high Coulombic efficiency close to 99.5% were expressed after 100 cycles, and their capacity preceded the theoretical value of graphite as well (279 mA h g^−1^) (Figure 8c).

Additionally, processing MXenes to 3D porous structures such as aerogels provides effective solution to the large volume stress induced by electroplating, which is a potent way to improve the electrochemical performances of PIB electrodes.^[^ ^172^, ^173^ ^]^ Shi et al. dipped commercial flexible melamine foam (MF) with a continuous macroporous structure into highly concentrated MXene ink and freeze‐dried the construct to form a flexible and light‐weighted 3D MXene–MF composite with a specific surface area of 24 m^2^ g^−1^. The molecular chains of MF (NH_2_, NH, and imine) and the abundant surface groups of MXene (─OH and ─F) could form strong bonds and ensure the compact attachment between MXene nanoflakes and MF skeleton during drying process. This unique bonding endowed the 3D MXene‐MF with robust mechanical performance, and the MXene‐MF‐Li electrodes for PIBs could keep circulating for 3800 h (equivalent to 1900 cycles) at a high current density of 10 mA cm^−2^.

#### 3.3.3 Zinc‐Ion Batteries (ZIBs)

Metallic Zn anodes not only exhibit a high theoretical capacity of 820 mA h g^−1^ and an appropriate redox potential of −0.762 V versus SHE, but they are also advantageous in terms of their environmental safety, cost efficiency, nontoxicity, and simple fabrication process.^[^ ^174^, ^175^ ^]^ As with alkali metal ions, the largest obstacles Zn‐based anodes face are the extreme volume change and the growth of Zn dendrites during electroplating and exfoliation procedures, which will destroy the structure of electrodes and lead to the interfacial instability.^[^ ^176^ ^]^ 2D MXenes with open and porous structures can potently restrain the growth of Zn dendrites and relieve the volume variation as well. Simultaneously, their abundant surface functional groups endow them with hydrophilicity and insolubility, ensuring the electrode materials are both wettable and chemically stabile in aqueous electrolytes. These characteristics and advantages of MXene‐based anodes for ZIBs have already been proved by early investigations.^[^ ^177^ ^]^

Conventional anodes for ZIBs include manganese (Mn)‐based materials, vanadium (V)‐based materials, and Prussian blue analogues. Flexible electrodes prepared by the hybridization of these materials with MXenes can perform as superior candidate anodes for ZIBs to further improve their properties. As a typical example, the tunnel‐structured VO_2_ could be favorably combined with firm, flexible, and conductive MXene carriers to generate a composite film with fast ion/charge transport kinetics.^[^ ^178^ ^]^ Additionally, the tough VO_2_/MXene electrode expressed considerable flexibility and favorable mechanical strength even under large‐angle deformations, which could be folded to the four characters of “SCNU”.^[^ ^179^ ^]^ The assembled flexible quasi‐solid‐state ZIB maintained an intact structure and stable electrical performance at different bending angles, demonstrating an outstanding cyclic stability with 72.1% capacity retention after 2500 cycles (Figure 8d).

Similarly, the mixture of (NH_4_)_2_V_10_O_25_·8H_2_O (NHVO) and Ti_3_C_2_T*_x_* could also form a flexible composite film, and the assembled structure of alternating Ti_3_C_2_T*_x_* nanosheets and NHVO nanoribbons constructed an effective face‐to‐line connection. Additionally, the intercalation of NHVO nanoribbons into Ti_3_C_2_T*_x_* layers suppressed the self‐restacking of Ti_3_C_2_T*_x_* and facilitated ion transport. Benefiting from the synergistic effect of NHVO and MXene, the prepared ZIB expressed only a slight capacity attenuation of 7.9% even after 6000 cycles at 2.0 A g^−1^. Similar to other ion batteries, 3D MXene aerogels were also applied to increase the specific surface area of electrodes, which reduced the local current density and prevented the formation of Zn dendrites. Zhou et al. fabricated the 3D MXene/graphene hybrid aerogel, and then deposited Zn onto it to obtain a whole electrode.^[^ ^180^ ^]^ The aerogel electrode could withstand 180° folding without fractures and the assembled ZIB possessed prominent Coulombic efficiency of 99.67% after 600 cycles at a high current density of 10 mA cm^−2^. By further introducing the multiscale strategy into the design of rGO/MXene aerogel, Cheng et al. prepared a flexible and self‐healing Zn‐ion battery.^[^ ^181^ ^]^ The in‐plane nanopores generated by the chemical oxidation with hydrogen peroxide (H_2_O_2_) solution offered extra ion channels, while the microscale pores formed by the gas foaming technique with hydrazine hydrate (N_2_H_4_) supplied sufficient ionic active sites and electron channels. The Zn battery based on multiscale MXene aerogel presented an excellent energy density of 156.8 µWh cm^−2^ and the wearable promising.

In summary, the utilization of flexible MXene‐based composites has emerged as a highly promising avenue for application in LIBs, LSBs, and their emerging NLIB counterparts. To address inherent challenges such as self‐stacking tendencies in MXenes and the volumetric fluctuations experienced in metal batteries, the fabrication of heterostructured MXene films and the development of porous MXene foams have emerged as universal strategies. Additionally, innovative techniques for preparing MXene‐based electrodes have been proposed in various metal battery systems, addressing specific issues such as the deposition of additional metals to serve as adhesive layers, thus mitigating exfoliation concerns and minimizing the growth of branch‐like crystals. Nevertheless, further investigations remain imperative to fully harness the potential of flexible MXenes in battery applications and to establish more optimal combinations therein.

## 4 Application of MXene‐Based Flexible Materials for Supercapacitors

Due to their high power density, fast and stable charging–discharge capability, and long cycling lifetime, supercapacitors (SCs) are widely utilized in vehicle start–stop systems and are potential candidates for future energy storage devices with high safety. The exhibited electrochemical superiorities of MXenes (such as the metallic‐like conductivity, large specific surface area, and abundant terminal groups) are the key driver for the development of MXene‐based SCs. Furthermore, in comparison with conventional electrode materials that tend to exhibit stiffness, rigidity, and brittleness, MXene‐based composites instead possess flexibility and durability, capable of meeting the demands of repeatedly bending, twisting, folding, and even stretching in flexible and wearable energy storage systems. Therefore, MXene‐based SCs present significant application values in next‐generation flexible electronics and smart fabrics. In this section, MXene‐based SCs of various types, including fibrous, paper‐shaped, and 3D structured forms, are introduced and their fabrication and applications are presented.

### 4.1 Fibrous MXene‐Based Supercapacitors

**Figure 9:**
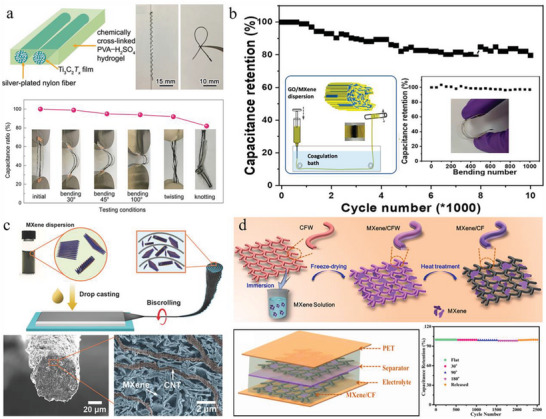
Fibrous MXene‐based SCs and their properties. a) The all‐solid‐state fiber‐shaped SC with MXene‐coated silver‐plated nylon fibers and the properties of MXene‐based fibers under different deformations. Reproduced with permission.^[^ ^74^ ^]^ Copyright 2017, Wiley‐VCH. b) Illustration of the preparation for GO/MXene fibers and the capacitance retention during charge–discharge cycles and bending cycles, respectively. Reproduced with permission.^[^ ^80^ ^]^ Copyright 2020, American Chemical Society. c) The biscrolled MXene/CNT fibers for SCs and cross‐sectional SEM images with different magnifications. Reproduced with permission.^[^ ^31^ ^]^ Copyright 2018, Wiley‐VCH. d) Flowchart of MXene/biomass derived carbon fibers (MXene/CFs) and the corresponding symmetric SC with cyclic performances. Reproduced with permission.^[^ ^182^ ^]^ Copyright 2021, Elsevier.

Fibrous SCs have shown extensive promise for wearable electronics. 1D fiber‐shaped MXene electrodes can be fabricated by coating the MXene‐contained colloids onto flexible fiber substrates. Hu et al. for the first time employed silver‐plated nylon fibers as support architectures and fabricated all‐solid‐state Ti_3_C_2_T*_x_* MXene SCs via drop‐casting.^[^ ^74^ ^]^ Due to the relatively low series resistance and high mechanical strength, the fabricated supercapacitors exhibited a high areal capacitance of 328 mF cm^−2^ with almost no decrease after 10 000 charge–discharge cycles. One single supercapacitor was capable of suspending an object with a mass of 200 g without incurring damage and maintained above 80% of the initial capacitance while bending, twisting, and even knotting, demonstrating impressive mechanical stability and flexibility (**Figure** **9**a). Similarly, Yun et al. applied a bottom‐up LbL assembly technique for the preparation of MXene‐based wire‐shaped supercapacitors.^[^ ^183^ ^]^ Activated carbon yarns (ACYs) were chosen as flexible substrates, on which positively charged rGO functionalized with poly(diallyldimethylammonium chloride) (rGO‐PDDA) and negatively charged Ti_3_C_2_T*_x_* MXene nanosheets were alternatively deposited with great conformality. The assembled wire‐shaped electrode yielded a high volumetric capacitance of 2193 F cm^−3^, manifesting a 240% enhancement compared with the uncoated ACYs. In addition, CV and charge–discharge curves of the LbL‐coated ACY with solid gel electrolyte showed no obvious change even at a bending radius of 3 mm, and the capacitance retention reached 90% after 200 cyclic bending tests.

However, the coating methods in these reports limited the MXene loading content, and thus constrained the electrochemical performance of 1D SCs. Further, the interfacial bonding between MXenes and fibrous substrates is weakened after several deformations, diminishing the devices’ flexibility and long‐term durability. Alternatively, direct MXene‐incorporating flexible hybrid fibers can be prepared by mixing active MXenes with other functional materials in a proper ratio and more consistently provide superior electrochemical performances and flexibility. As an example, GO with a high aspect ratio has been mixed with MXene to form a spinnable aqueous dispersion. After reduction and wet spinning, the mixed rGO/MXene fiber electrode reached a useful balance of conductivity (743.1 S cm^−1^), mechanical strength (110.7 MPa), and toughness (3.8 MJ m^−3^) with an MXene concentration of 60 wt%.^[^ ^80^ ^]^ The assembled fiber‐shaped SCs (FSCs) expressed a large areal capacitance of 550.96 mF cm^−2^, an attractive energy density of 9.85 mW h cm^−3^ at a power density of 8.8 mW cm^−2^ (7.1 W cm^−3^), and demonstrated relatively small changes of capacitance even after 10 000 charge–discharge cycles or 1000 bending cycles (15% degradation and 5% fluctuation, respectively) (Figure 9b). When the MXene content in the rGO/MXene fiber electrode was increased to 90%, the volumetric capacitance and the conductivity were further raised to 586.4 F cm^−3^ (nearly twice than that of the 60% MXene fiber) and 2.9 × 10^4^ S cm^−1^, while the tensile strength was relatively weakened.^[^ ^184^ ^]^ Utilizing the spinnable PEDOT:PSS conductive polymer as the binder could also ensure the wet‐spinnability of MXene‐based dispersions, and a hybrid fiber with 70% MXene possessed a high conductivity of 1489 S cm^−1^ and a high volumetric capacitance of 614.5 F cm^−3^.^[^ ^82^ ^]^ The assembled independent FSC reached an energy and power density of ≈7.13 mW h cm^−3^ and ≈8249 mW cm^−3^, respectively. Also, after wrapping on a prestretched silicone substrate, the FSCs could still withstand 100% tensile strain with 96% capacitance retention. By the deliberately designed biscrolling method, loading of up to 98% MXene on a CNT scaffold was realized through the formation of a helically structured fiber with interstitial space, which aided electrolyte penetration.^[^ ^34^ ^]^ The mixed flexible MXene/CNT fiber delivered an areal capacitance up to 3188 mF cm^−2^ at 2 mV^−1^ and still maintained 2506 mF cm^−2^ (78.6%) as the current density increased ten times, proving its outstanding rate capability. The asymmetric yarn‐shaped supercapacitor (aYSC) prepared by pairing the MXene/CNT anode with the RuO_2_/CNT cathode possessed a wide potential window of 1.5 V and an excellent energy density of 61.6 mW h cm^−3^, and its CV curve presented an almost 100% capacitance retention after 1000 bending cycles at 90° (Figure 9c).^[^ ^31^ ^]^ Also, this reported aYSC demonstrated a potential conformal energy solution for powering electronics. The fabric woven by 100 threads of aYSCs with an area of 1 cm^2^ could provide an energy of ≈0.44 mW h, which is almost equivalent to the one‐day energy consumption of a 34 µW transmitter. In addition, Wang et al. reported a confined hydrothermal strategy to produce rGO/MXene composite fibrous electrodes.^[^ ^185^ ^]^ Due to the synergistic effect between relatively large rGO and small MXene, the prepared electrode was flexible enough to be woven into a knitted glove and showed a volumetric capacitance of 345 F cm^−3^ at 0.1 A g^−1^ with only 5% MXene loading, retaining 95% of its original capacitance after a month. The rGO/MXene composite SC demonstrated a volumetric energy density of 30.7 mW h cm^−3^.

Knitting is an effective way to produce MXene‐based energy storage textiles with flexible fiber‐shaped MXene composites. Ten‐meter‐long MXene‐coated yarns prepared by repeatedly coating the MXene solution on cotton yarns were knitted into textile SCs via computer aided design.^[^ ^75^ ^]^ The obtained energy storage textile provided a high areal capacitance up to 707 mF cm^−2^ in 1 m H_3_PO_4_, and retained 100% capacitance with ≈100% Coulombic efficiency after 10 000 charge–discharge cycles. These superior electrochemical performances introduce the possibility for the large‐scale production of 3D‐knitted textile supercapacitors.

Another strategy to introduce MXenes into textile is directly attaching them onto carbon‐based skeleton substrates with outstanding mechanical performance, such as carbon cloths and carbon wipers. MXene/biomass derived carbon fibers (MXene/CFs) were obtained by immersing a commercial cotton fiber wiper into the MXene solution and pyrolyzing the resulting textile with a high‐temperature process.^[^ ^182^ ^]^ SEM imaging revealed that the original smooth CF became rough after the introduction of MXene but still retained its loose and porous structure, which indicated the uniform coating of MXene on CF and the successful formation of a skin/skeleton‐like porous heterostructure textile that supplied abundant channels for electrolyte penetration and fast ion diffusion. The prepared all‐solid‐state symmetric supercapacitor manifested an outstanding stability, whereby the capacitance only decreased by 2.3% after 2500 cycles at different bending angles (Figure 9d). This approach has also been suitable for the large‐scale fabrication of asymmetric supercapacitors (ASCs).^[^ ^186^ ^]^ The MXene decorated N‐doped carbon fiber textile (NCFT) was chosen as the anode material, and the chemically oxidized NCFT was chosen as the cathode. The integrated ASC showed an extended voltage potential of 1.6 V and an outstanding energy density of 277.3 µW h cm^−2^ (equivalent to 16.3 W h kg^−1^). Additionally, capacitance retention of up to 90% was exhibited after 10 000 bending cycles at 180° or 30 000 charge–discharge cycles at 50 mA cm^−2^, demonstrating the potential of ASCs in long‐term wearable electronics. Furthermore, the knitted structure of clean wipers endowed their composites with excellent stretchability, and the Ti_3_C_2_T*_x_* MXene/clean wiper supercapacitor prepared by drop‐casting not only provided a favorable tensile strength of 20.1 MPa and a prominent areal capacitance of 118 mF cm^−2^, but also inherited the stretchability to withstand up to 40% tensile strain while maintaining a stable capacitance.^[^ ^61^ ^]^ In addition to attaching pure MXene onto textile surfaces, the in situ growth of MXene/MWCNT composite on CC (denoted as MWCNT‐MXene@CC) was an effective solution to further enhance electrochemical performances.^[^ ^187^ ^]^ MXene nanoflakes were attached to CC by a simple immersion process, and MWCNTs were grown through low‐pressure CVD, applying acetylene (C_2_H_2_) gas as the carbon source. The in situ grown MWCNT and MXene composite exhibited relatively decreased internal resistance and prevented the self‐aggregation of MXene layers. With a scan rate of 5 mV s^−1^, the MWCNT–MXene@CC reached a high areal capacitance of 114.58 mF cm^−2^, which was 62% higher than that of pristine MXene. The resistance change was less than 5% during 2000 bending cycles, showing outstanding mechanical stability.

The exterior coating and growth of MXene is constrained by the available surface area of substrates and thus prohibits the intrinsic improvement of MXene loading capability and overall performance. Electrospinning a mixed solution of MXene and other active materials has served as an effective way for large‐scale fabric fabrication, and can also alleviate some of these problems. For example, the MXene nanoflakes loaded with FeCo_2_S_4_ nanoparticles were utilized as a component for the spinnable material.^[^ ^90^ ^]^ In situ carbon coating technology was applied to embed sulfide‐loaded MXene into electrospinning fibers, and a thermally induced phase separation process was employed to introduce meso/macropores and generate porous carbon nanofibers (or PCNFs). This special structure provided channels for fast and continuous electrolyte diffusion as well as the required buffering for local volume variation during charging–discharging process. The optimized FeCo_2_S_4_/MXene/PCNF fabric electrode manifested a high specific capacitance of 446.4 mA h g^−1^ and an impressive rate capability of 73.87% capacitance retention (from 1 to 20 A g^−1^). Additionally, 98.1% of the initial capacitance was retained even after 10 000 charge–discharge cycles, without obvious changes in the morphology of the fabric electrode as observed by SEM imaging.

### 4.2 Paper‐Shaped MXene‐Based Supercapacitors

MXene papers prepared by vacuum filtering and natural deposition have occupied a large proportion of MXene‐based electrodes for SCs. To improve electrochemical performances of MXene‐based paper electrodes in SCs, two common‐used strategies are considered. The first strategy is to introduce other electrode materials into MXenes to prepare composite flexible films.^[^ ^4^, ^192^ ^]^ According to our discussion in Section 2.1, the unique accordion‐like structure of MXenes contributes to their high conductivity and relatively short ion diffusion distance, which highlight them as ideal materials for high‐performance SCs. Unfortunately, their self‐aggregation during film production processes and cyclic tests limits their practical capacity and long‐term durability. Combining MXenes with other active electrode material might express synergetic effects to overcome shortcomings of both materials and improve entire performances. The doping of heteroatoms or functional groups has been verified to be another effective approach to enhance the properties of MXene‐based paper electrodes, since it modifies the surface of MXene nanosheets and ameliorate physical and chemical properties.^[^ ^193^, ^194^ ^]^ In this section, two typical types of SCs, denoted as electric double layer capacitors (EDLCs) and pseudocapacitors (PCs), constructed by the combination of MXenes and other electrode materials, and the introduction of terminations are separately discussed.

#### 4.2.1 MXene‐Based Films for EDLCs

**Figure 10:**
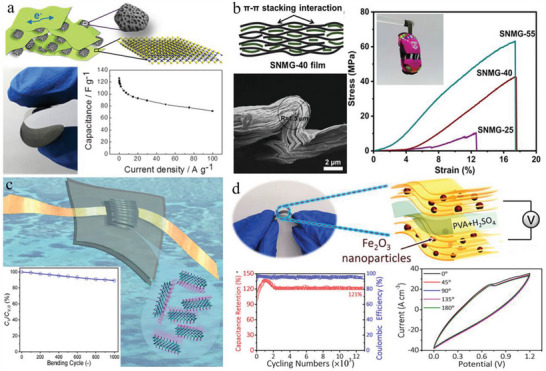
MXene‐based flexible papers and films for SCs and their enhanced properties. a) The flexible MXene film with porous AC particles and the mechanical/electrochemical performances for AC/MXene electrodes. Reproduced with permission.^[^ ^188^ ^]^ Copyright 2018, American Chemical Society. b) The rGO/MXene film with a bending radius of 1.5 µm and the enhanced fracture strain. Reproduced with permission.^[^ ^189^ ^]^ Copyright 2021, Wiley‐VCH. c) Illustration of an EDLC with BP‐expanded MXene electrodes and the high capacity retention during bending cycles. Reproduced with permission.^[^ ^190^ ^]^ Copyright 2020, Elsevier. d) Views of the Fe_2_O_3_‐doped MXene PC and the electrochemical performances with repeated cycles and different bending angles. Reproduced with permission.^[^ ^191^ ^]^ Copyright 2020, American Chemical Society.

The energy storage mechanism of EDLCs is electrostatic ion absorption at the interface between electrolyte and electrode. Their electrode materials, including carbon materials and other nonmetallic materials with large specific surface area and high conductivity, possess relatively low density and limited contact area with electrolytes.^[^ ^195^ ^]^ Fortunately, the doping of these materials into MXenes could always supply extra sites for ion transport as well as illustrating enhanced flexibility and stability. Early in 2014, Gogotsi's group creatively reported MXene‐based composites that reach high capacitance.^[^ ^196^ ^]^ Their strategy of alternatively filtering Ti_3_C_2_T*_x_* MXene and CNT to prepare the sandwich‐like Ti_3_C_2_T*_x_*/CNT paper was also demonstrated to be adaptable for doping other carbon components into MXene‐based film, including but not limited to 0D onion‐like carbon and 2D rGO. With only 5% CNT content, the flexible MXene/CNT electrode provided a high capacitance density of 199 F g^−1^ at a large current density of 500 A g^−1^ and a 92% capacitance retention after 10 000 charge–discharge cycles.^[^ ^197^ ^]^ Meanwhile, the 25 mg hybrid film could withstand 720 times its own weight and exhibited the flexibility to be arbitrarily bent, rolled, or folded. Similarly, Wang et al. reported a Co@N‐CNT/MXene film with an excellent specific capacitance of 97.7 F g^−1^, which also possessed an astonishing capacitance retention of 92.7% and a Coulombic efficiency of 99.7% after 85 000 cycles.^[^ ^35^ ^]^ The roughly superimposed CV curves at different bending angles (from 45° to 180°) also demonstrated its flexibility and stability to retain original levels of capacitance. MXene hybrids with low loading could also replace conventional materials, such as PVDF, PTFE, etc., as the binder for activated carbon (AC) particles in the composite film, which provided both flexibility and extra capacitance contribution.^[^ ^188^ ^]^ The AC particles with abundant microporous structures were encapsulated between MXene layers and provided additional active sites, remarkably increasing the interlayer distance of MXene nanosheets to benefit electron transport and electrolyte penetration (**Figure** **10**a). As a result, the bendable AC/MXene electrode supplied an excellent specific capacitance up to 126 F g^−1^ (at 0.1 A g^−1^) and an attractive capacitance retention of 57.9% at the high current density of 100 A g^−1^.

rGO is another common doping material that can insert between MXene layers and establish a strong interaction by electrostatic self‐assembly with MXene in a dispersion. Only 5 wt% of rGO increased the interlayer distance of Ti_3_C_2_T*_x_* MXene from 1.3 to 1.5 nm.^[^ ^198^ ^]^ Concomitantly, the prepared electrode exhibited a noticeable volumetric capacitance of 1040 F cm^−3^ (at 2 mV^−1^) as well as a capacitance retention of 61% (at 1 V^−1^), while holding an ultrahigh volumetric energy density of 32.6 W h L^−1^. As the size and doping ratio (up to 40%) of rGO increased, structural changes occurred in the composite.^[^ ^189^ ^]^ Large sized rGO flakes functioned as the scaffolds for loading smaller MXene sheets and strengthening the Ti─O─C covalent bonding, which contributed to the ordered LbL alignment of composite flakes and the large‐area continuous fabrication of the flexible film through a blade coating process. Owing to the relatively low conductivity of rGO, the specific capacitance and power density (698.5 F cm^−3^ and 22.3 W h kg^−1^) were slightly decreased compared to lower rGO‐loading samples. By contrast, the flexible rGO/MXene film exhibited a great improvement of mechanical stability, including a high tensile strength of 45 MPa (compared with 10–20 MPa of pure MXenes) and the bendability at 1.5 µm bending radius (Figure 10b). Introducing a conjugated molecule like 1‐aminopyrene‐disuccinimidyl (AD) suberate into rGO/MXene hybrids could form π–π bridging between adjacent rGO flakes, which further improved the mechanical performances of the composite paper.^[^ ^199^ ^]^ The resulting electrode retained a toughness of ≈42.7 MJ m^−3^ with a prominent failure strain of 12% and possessed an incredible tensile strength of ≈699.1 MPa. Furthermore, the integrated MXene‐functionalized graphene SC preserved ≈98% of its initial capacitance after 17 000 bending cycles at 180°, and reached a remarkable energy density of 39.4 W h L^−1^ and an unchanged CV curve under 180° bending with H_2_SO_4_ mediation.^[^ ^200^ ^]^

Other than carbon materials, emerging inorganic nonmetallic materials with favorable conductivity have also been investigated and introduced into MXenes. BP flakes that possess a natural puckered structure, relatively high packaging density (2.69 g cm^−3^), and good conductivity (300 S m^−1^) was introduced to MXene to expand its electrolyte diffusion channels.^[^ ^190^ ^]^ The Ti─O─P bonds formed between BP and MXene facilitated effective charge transfer process as well. As a result, the quasi‐solid‐state SC with the BP‐expanded MXene electrode demonstrated a high specific capacitance of 640 F cm^−3^ and an excellent rate performance (86.4% capacitance retention from 2 mV s^−1^ to 1 V s^−1^) in a neutral Na_2_SO_4_ electrolyte, and still maintained 89.2% of its initial capacitance under 1000 bending cycles at 90° (Figure 10c).

#### 4.2.2 MXene‐Based Films for PCs

As for PCs that perform charge–discharge processes by rapid Faraday reaction on the surfaces of electrodes, the introduction of MXenes benefits the adherence of common‐used electrode materials, such as small‐sized metallic compounds and conductive polymers, and thus depresses their volume change as well as ensures flexibility. At the same time, the inserted metallic compounds can increase the interlayer space of MXenes and provide more electrochemically active sites, constructing a synergistic facilitation. For instance, doping Fe_2_O_3_ nanoparticles into MXene papers supplied more charge storage sites, which resulted in obviously increased integral area in CV curves calculated to an outstanding capacitance of 2607 F cm^−3^ (584 F g^−1^).^[^ ^191^ ^]^ In contrast with the pure MXene or pure Fe_2_O_3_ electrodes, the Fe_2_O_3_‐anchored MXene electrode had a wider potential window from −0.5 to 0.5 V and a 121% capacitance retention after 13 000 charge–discharge cycles. The assembled symmetric capacitors illustrated almost identical CV curves at 0°–180° bending angles, demonstrating advantageous mechanical stability and the prospect for wearable applications (Figure 10d). Similarly, a Prussian blue analog made of 3D Ni–Fe oxide was mixed with MXene at a mass ratio of 1:4 to produce a flexible composite, reaching an areal capacitance of 1038.43 mF cm^−2^ with up to 82.23% capacitive retention.^[^ ^201^ ^]^ The all‐solid‐state supercapacitor consisting of the composite electrodes, PVA/LiCl electrolyte and separator exhibited the excellent capacitance retention of 85.5%, 90.9%, and 89.9% (while the current density increased from 0.2 to 1.0 mA cm^−2^, processed 10 000 charge–discharge cycles and 50 bending cycles at 90°, respectively). The outstanding rate performance as well as excellent electrochemical and mechanical stability of the heterostructured MXene‐based film was unachievable by pure Prussian blue. Meanwhile, the MXene‐based paper with a high loading percentage of MoO_3_ nanobelts (with a mass ratio of 2:8) had four pairs of redox peaks, which contributed to the volumetric capacitance up to 1836 C cm^−3^ and the energy density up to 39.2 Wh L^−1^ as well as retaining 94.2% capacitance during 10 000 charge–discharge cycles.^[^ ^202^ ^]^

Functionalized polymers illustrate a similar energy storage mechanism to metallic compounds, while their differences include better flexibility, relatively poor electrical conductivity, and unsatisfactory rate performance. Gogotsi's group for the first time reported the Ti_3_C_2_T*_x_*/PVA MXene–polymer composite, which possessed a superior conductivity of 2.2 × 10^4^ S m^−1^ and a specific capacitance of ≈530 F cm^−3^.^[^ ^203^ ^]^ When the doping content of PVA was increased to 60%, the flexible paper showed an improved tensile strength of 91±10 MPa, which was much larger than that of the pure Ti_3_C_2_T*_x_* paper (22±2 MPa) and the pure PVA film (30±5 MPa), demonstrating the robust mechanical interactions between these two materials. Moreover, a higher volumetric capacitance (873 F cm^−3^) and a balanced volumetric/areal energy density (20.9 Wh L^−1^ and 90.3 µWh cm^−2^) were obtained by assembling intrinsically conductive polyaniline (PANI) nanoparticles onto the Ti_3_C_2_T*_x_* MXene.^[^ ^204^ ^]^ The hybrid Lig@Ti_3_C_2_T*_x_*/PANI@Ti_3_C_2_T*_x_* film prepared with the mixture of lignosulfonate (Lig)‐loaded Ti_3_C_2_T*_x_* nanoflakes (Lig@Ti_3_C_2_T*_x_*) and PANI‐loaded Ti_3_C_2_T*_x_* nanoflakes (PANI@Ti_3_C_2_T*_x_*) at a mass ratio of 1:1 illustrated the enhanced tensile strength and vertical‐plane tensile strength of 53.7 and 0.58 MPa (while those of PANI@Ti_3_C_2_T*_x_* film were 33.2 and 0.28 MPa), respectively.^[^ ^205^ ^]^ Meanwhile, an outstanding volumetric capacitance of ≈959 F cm^−3^ and a high energy density of 33.3 W h L^−1^ were also observed. These superiorities were mainly ascribed to the abundant phenol groups of the nonconductive Lig, which contributed to both its hydrogen bonding with Ti_3_C_2_T*_x_* and its high pseudocapacitance. As the result of the synergy, the composite film could avoid interlayer exfoliation and maintain integrity under various mechanical stresses, including bending, twisting, and arbitrarily folding. The introduction of another commonly used conductive polymer, denoted as polypyrrole (PPy), could also supply additional flexibility and stability by preventing the MXene from self‐aggregation.^[^ ^206^ ^]^ Furthermore, the innovative strategy to introduce the “dual spacers” of PPy and ionic liquid (IL)‐based microemulsion particles avoided the swelling of PPy during operation and simultaneously provided sufficient area accessible to ions. The interlayer space of PPy–MXene–IL–mic film was clearly expanded to 1.90 nm, which was far larger than 1.29 nm of the pure Ti_3_C_2_T*_x_* MXene film. The as‐prepared hybrid electrode possessed an excellent gravimetric energy density of 31.2 W h Kg^−1^ at 1030.4 W kg^−1^ and illustrated a capacitance retention and Coulombic efficiency of 91% after 2000 cycles.

#### 4.2.3 Termination‐Introduced MXene Films for SCs

Referring to the discussion in Section 2.1, Ti_3_C_2_T*_x_* MXenes prepared by typical HF etching is loaded with rich ─OH, ─F terminations and thus is macroscopically hydrophilic, leading to the increased accessibility of water‐dissolved oxygen and fast oxidation. Liao et al. employed a H_2_/Ar protected annealing treatment to remove the hydrophilic terminations and to realize sulfur‐ and nitrogen‐doping in the prepared S,N‐MXene/rGO hybrid film.^[^ ^189^ ^]^ The doped flexible composites exhibited a proper water contact angle (91° compared with 94° of the S,N‐MXene film and 86° of the MXene/rGO film), leading to improved water‐resistant capability and oxidation stability with undiminished flowability for film preparation. The obtained electrode demonstrated a nearly 98% capacitance retention after 30 000 charge–discharge cycles, and could be preserved under ambient conditions or the H_2_SO_4_ electrolyte for over 100 days, possessing a potential for industrial‐scale applications. Similarly, with a protective hydrothermal method, the doping of N atoms and the controlled growth of TiO_2_ were realized in the production process that endowed the flexible N‐doped heterostructured Ti_3_C_2_T*_x_*/TiO_2_ film with rolling deformation capability.^[^ ^193^ ^]^ The N‐doped MXene paper electrode provided an ultrahigh specific capacitance of 2194.33 mF cm^−2^ (918.69 F g^−1^), which surpassed most of the previously reported MXene electrodes with heteroatom doping. This unique performance was partly attributed to the formation of wrinkles and large electrostatic repulsions between MXene interlayers induced by N‐doping, which as a result prevented the MXene from restacking and ultimately facilitated the electrolyte diffusion. Further, N‐doping provided additional active sites for redox reactions and higher pseudocapacitance for film electrodes. An identical effect was observed when Mn^2+^ was intercalated into the MXene‐based electrode by adding MnCl_2_ into the MXene solution.^[^ ^194^ ^]^ The introduced Mn ions tended to bond with oxygen‐containing terminal groups, which linked the Ti_3_C_2_T*_x_* nanosheets and supplied speedy charge transport channels. The Mn^2+^‐doped flexible MXene films exhibited a high conductivity of 4268 S m^−1^, which was over twice that of the pristine MXene. Inspired by the conventional synthesis mechanism that introduced hydrophilic groups, Guo et al. first employed an LiF–H_2_SO_4_ solution as an alternate etching agent to HF in order to instead impart bulky ─SO_4_ terminations.^[^ ^207^ ^]^ Both SEM imaging and DFT calculation indicated the successful introduction of ─SO_4_ termination and the expansion of Ti_3_C_2_T*_x_* interlayer spaces without conductivity decrease. Not surprisingly, the modified MXene film possessed a high areal capacitance of 1399.0 mF cm^−2^ and an outstanding durability to endure diversified deformation, including bending, twisting, rolling, and even folding. No significant capacitance loss of the SC was observed after 17 200 charge and discharge cycles, which demonstrated its excellent electrochemical capability, flexibility, and long‐term stability and indicated the validity of sulfate intercalation.

### 4.3 3D Structured MXene‐Based Supercapacitors

Considerable research and publications have been devoted to 3D structural innovations to diminish the natural self‐aggregation and restacking in MXene electrodes during preparation procedures in order to achieve higher volumetric and areal capacitance. Zhang et al. selected commercial melamine foam as the template to absorb Ti_3_C_2_T*_x_*/GO nanosheets and prepared a Ti_3_C_2_T*_x_* MXene/rGO/carbon hybrid foam.^[^ ^208^ ^]^ The porous structure benefited the absorption and diffusion of the electrolyte and led to an up to 276 F g^−1^ gravimetric capacitance of the electrode. The annealed foam could also withstand up to 60% compression strain and recover without any shape change and illustrated deformability to be bent or twisted. Similarly, the foam electrode prepared with the carbon foam template hold a high volumetric capacitance of 3162 mF cm^−3^ and an almost unchanged structure after 500 compression–release cycles under 60% strain.^[^ ^209^ ^]^ The CV and GCD curves demonstrated an almost constant capacitance performance in the solid‐state foam SC under different compression conditions, indicating no electrolyte leakage.

**Figure 11:**
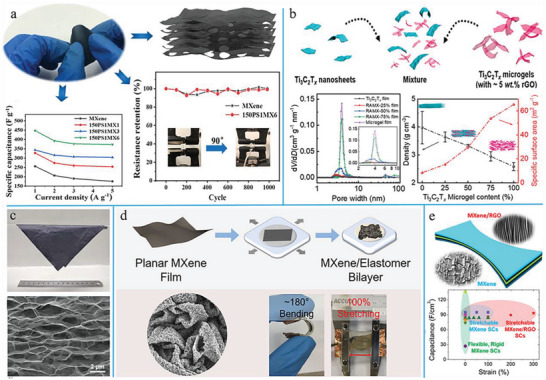
3D structured MXene‐based SCs and their unique features. a) The MXene‐based 3D porous network prepared with PS sacrificial materials and the electrical properties of different porous MXene films and during bending cycles. Reproduced with permission.^[^ ^210^ ^]^ Copyright 2021, American Chemical Society. b) The mesoporous Ti_3_C_2_T*_x_* MXene electrode with the mixture of Ti_3_C_2_T*_x_* nanosheets and Ti_3_C_2_T*_x_* microgels and the properties related to microgel content. Reproduced with permission.^[^ ^211^ ^]^ Copyright 2021, Wiley‐VCH. c) The porous MXene electrode for SCs prepared by synchronously reducing and self‐assembling followed with freeze‐drying. Reproduced with permission.^[^ ^212^ ^]^ Copyright 2021, Wiley‐VCH. d) Preparation process, SEM image, and deformation photographs of the crumpled MXene electrode. Reproduced with permission.^[^ ^213^ ^]^ Copyright 2018, American Chemical Society. e) The MXene/rGO SCs with advanced tensile strain and capacitance. Reproduced with permission.^[^ ^214^ ^]^ Copyright 2021, American Chemical Society.

Alternatively, the insertion and subsequent removal of PS, PMMA or other microspheres in MXene films was a common‐used method to construct the 3D porous network with minimum influence on its mechanical behavior.^[^ ^215^ ^]^ Additionally, the controllable optimization of porosity and mesopore size in porous MXene films was realized by regulating the doping ratio and dimension of PS microspheres.^[^ ^85^, ^210^ ^]^ The optimized flexible MXene electrode with porous structure achieved a high gravimetric capacitance of 506 F g^−1^ as well as a volumetric capacitance of 759 F cm^−3^ at 0.5 A g^−1^ while maintaining a satisfactory gravimetric capacitance of 380 F g^−1^ with a raised current density of 20 A g^−1^, demonstrating desirable rate behavior. The electrode also delivered an almost unchanged electrical performance during 1000 bending cycles at 90° (**Figure** **11**a). Analogously, Fe(OH)_3_ nanoparticles were also selected as sacrificial materials.^[^ ^216^ ^]^ The removal of nanoparticles and surplus terminal groups as well as the raised proportion of Ti atoms was realized by HCl etching and low‐temperature calcination at 200 °C under the premise of preserving the hydrophilicity of the MXene. In comparison with the PS templating strategy, this method provided higher pseudocapacitance for the bendable MXene film and correspondingly expressed a higher volumetric capacitance (1142 F cm^−3^ at 0.5 A g^−1^) and better rate behavior (828 F cm^−3^ at 20 A g^−1^).

In addition to direct templating, functional materials are also employed to construct a 3D porous network between MXene nanosheets. By reassembling Ti_3_C_2_T*_x_* nanosheets with Ti_3_C_2_T*_x_* microgels and removing interstitial water, mesopores with the size of 3–5 nm were introduced into the 3D MXene network (Figure 11b).^[^ ^211^ ^]^ The soft microgels could suppress the self‐aggregation of MXene nanosheets and assist with the formation of densely packed MXene electrodes. Further, the controlled optimization of density and specific surface area was accomplished by regulating the proportion of microgels in the MXene. The optimized flexible film realized rapid ion transfer and correspondingly delivered a high specific capacitance and energy density with a well‐maintained rate property, which decreased the overuse of electrolyte. In addition to these efforts, Chen et al. proposed a MXene‐based 3D network established with CNF and porous carbon (PC), providing abundant pores from microscale to macroscale while also facilitating charge storage and ion diffusion processes.^[^ ^217^ ^]^

Furthermore, more advanced techniques have been investigated and developed to form 3D structured MXenes. Based on a chain reaction, Miao et al. deliberately put forward an innovative self‐propagating process to prepare porous structural MXene within only 1.25 s.^[^ ^218^ ^]^ The as‐prepared MXene/GO hybrid film was placed in contact with a 300 °C heating plate in a glovebox filled with argon. During this process, the GO was reduced to rGO accompanying the releasing of surface functional groups and the formation of substantial gas volume, which led to the formation of a porous structure in the composite electrode. The SC assembled with the fluffy MXene/rGO electrode in 3 m H_2_SO_4_ electrolyte possessed an outstanding gravimetric capacitance of 329.9 F g^−1^ and a 90% capacitance retention after 40 000 charge cycles. Zhao et al. fabricated a bendable MXene porous film by synchronously reducing and self‐assembling the MXene suspension onto a Zn substrate followed by freeze‐drying.^[^ ^212^ ^]^ SEM images indicated the obviously decreased thickness and the retained cross‐linked structure of the MXene film during drying process (with a proper drying time less than 3 h), which ensured efficient charge transmission (Figure 11c). This method could be extended to the scalable assembly of interdigital SCs, and the devices possessed rectangular CV curves even at a high scan rate of 5 V s^−1^ as well as a linear relationship between currents and scan rates, demonstrating satisfying capacitance and rate performance. Compared with vacuum‐assisted filtration, the freeze‐casting strategy could generate 3D pores between MXene layers with the expansion and sublimation of ice templates. The introduced 1D bacterial cellulose (BC) or CNT could bridge between MXene nanosheets, which provided larger interlayer spaces and correspondingly established a more developed porous structure after the sublimation of ice crystals.^[^ ^219^, ^220^ ^]^ Specifically, the prepared MXene/BC electrode with BC connections delivered an ultrahigh specific capacitance (416 F g^−1^, 2084 mF cm^−2^), and the asymmetric SC assembled with the MXene/BC anode and a PANI/BC cathode illustrated a wide potential window of 1.4 V, a superior energy density of 252 µWh cm^−2^, and almost unchanged CV curves under different bending angles from 0° to 180°. Yang et al. further described that the optimum assembly configuration of 2D materials was vertically aligned to the substrates, which enabled orientation‐specific ion transportation and thus achieved thickness‐independent performances.^[^ ^69^ ^]^ Based on this hypothesis, they reported a freeze‐assisted tape casting process that provided a vertical temperature gradient for the directional growth of ice crystals by careful reduction of the temperature of the casting bed. The growth of ice crystals induced a solid–liquid interfacial force, which separated the MXene nanosheets from each other and concentrated them between crystals to construct a vertical alignment. MXene films with different thickness, from 150 to 700 µm, were effectively prepared and were all shown to possess vertical alignment and interlinked structures via SEM imaging. Consequentially, all electrodes exhibited similar gravimetric capacitances and rate performances at up to 3000 mV^−1^ regardless of the thickness. The vertically aligned electrode with a thickness of 300 µm delivered a 97.7% capacitance retention after more than 14000 charge–discharge cycles and provided bendability up to at least 90°, indicating the excellent electrochemical stability and flexibility. Recently, Wu et al. investigated the pure MXene material with dense yet porous 3D structure, induced by hydroiodic acid.^[^ ^221^ ^]^ A novel face‐to‐edge electrostatic assembly was proposed on the basis of the attractions between negatively charged MXene surfaces and positively charged MXene edges. Promisingly, the assembled MXene monoliths expressed an unparalleled specific capacitance of 18.6 F cm^−2^.

Beyond the improvement of electrochemical properties, special surface topographies have been fabricated and programmed to supply additional stretchability for MXene‐based electrodes.^[^ ^222^ ^]^ An MXene film doped with sodium alginate and carbon black was adhered onto a prestretched elastic substrate and formed a crumpled structure by harnessing interfacial instability during contraction and releasing.^[^ ^213^ ^]^ By regulating the cycling and directionality of this process, the programmable preparation of MXene‐based electrodes with different topological structures was achieved. The roughly superposed CV curves indicated that the stretchable MXene‐based flexible electrodes maintained a nearly unchanged specific capacitance at 180° bending and 100% tensile strain (Figure 11d), and also delivered a capacitance retention of 96% after 1000 stretching/releasing cycles at 50% strain. Similarly, the MXene/rGO electrodes with crumpled structures maintained stable properties when suffering 300% uniaxial or 200% × 200% biaxial strains, providing an up to 49 mF cm^−2^ (≈490 F cm^−3^, ≈140 F g^−1^) specific capacitance.^[^ ^223^ ^]^

Another strategy to improve film properties has been to prepare thinner MXene films. At a thickness of only ≈3 µm, pure MXene film could withstand at least 800% areal strain and preserve a high specific capacitance of ≈470 mF cm^−2^ (Figure 11e).^[^ ^214^ ^]^ In addition, the ultrathin MXene films also possessed unique cycling stability, retaining more than 90% of their initial capacitance after 1000 stretching–releasing cycles. These attempts to endow stretchability to MXene‐based film by the creation of topological structures still exhibited significant defects, including relatively low specific capacitance, unachievable high energy density, as well as the flaking and breaking of MXene layers during stretching cycles. Despite this, researchers still demonstrated the potential to employ MXene‐based electrodes for wearable and stretchable SCs, expanding the field for future wearable energy systems.

Analogous to the considerations in battery systems, the enhancement of flexibility, specific capacitance, and rate performance in MXene‐based flexible SCs involves strategies that are somewhat akin but exhibit notable distinctions. These strategies are categorized into three subsections based on the morphology of the electrodes. It is essential to acknowledge that MXene‐based electrodes with varying geometries necessitate unique fabrication and improvement techniques, each endowed with distinct applicability. For instance, the production of wearable electronics predominantly employs fibrous MXenes, albeit hindered by the presence of binding agents and other additives that impede electrical conductivity. Consequently, a central challenge revolves around augmenting the MXene content while concurrently bolstering adhesion between disparate components. By contrast, pure MXene papers are readily accessible, warranting an exploration of the effects stemming from the introduction of alternative active materials and specialized terminals. Moreover, the utilization of 3D structured MXene‐based electrodes introduces supplementary properties to supercapacitors, including compressibility, thickness‐independent capacitance, and stretchability. In sum, the development of MXene‐based flexible supercapacitors can be comprehensively approached by considering functionalities, morphologies, and preparation methods.

## 5 Conclusion and Outlook

The growth of energy storage devices has prompted the work of multidisciplinary researchers, and MXene‐based flexible materials rely on the associated design of material science, chemistry, and mechanics, which has spurred significant advancements in the fields of batteries and supercapacitors. In this progress report, we summarize recent advances in promising MXene‐based flexible materials for energy storage requirements, including the preparation methods and their applications in LIBs, LSBs, SIBs, PIBs, ZIBs, and SCs. Beyond, we thoroughly discuss the mechanisms for MXene‐based flexible materials to possess remarkable electrochemical performances and strain‐withstanding features and we focus on their applications in flexible energy electronics. In general, MXene‐based flexible materials can ameliorate the conductivity of electrodes, accommodate to large volume expansions, contribute additional energetic active sites, and facilitate effective ion transport.

Even though tremendous developments have been accomplished in MXene‐based flexible electrochemical devices, some essential challenges still remain unsolved.^[^ ^224^ ^]^ First, there has always been a trade‐off between the mechanical properties (such as toughness, strength, and flexibility) and the electrical/electrochemical performances in almost all the flexible electronics. The same issue exists in MXene‐based electrodes, which puts an impetus on researchers to pursue the most optimal material and structural configurations for maximum performance results. As for MXene‐based flexible materials, the meticulous design of complex structures, such as 3D structures, porous structures, Janus structures, and bioinspired structures has been beneficial for the further enhancement of the flexibility, toughness, and tensile/compressive strength, and have correspondingly possessed significant influences on their electrochemical performances.^[^ ^208^, ^214^, ^225^, ^226^ ^]^ For example, Ti_3_C_2_T*_x_* MXene foams with micro‐ and nanopores always have improved specific capacity and cyclic stability compared to MXene papers without any microstructures.^[^ ^210^ ^]^ Nonetheless, the predominant focus of current research endeavors predominantly revolves around experimental investigations aimed at elucidating the qualitative correlations existing between the 3D microstructures of MXene‐based materials, including interconnected micropores and crumpled topological structures, and their corresponding mechanical and electrochemical attributes. In light of this, it is imperative to advocate for a shift toward more fundamental inquiries, specifically involving theoretical mechanisms and simulation analyses, in order to establish quantitative frameworks that facilitate a deeper understanding of these interrelationships.

Second, MXenes present poor stability against oxidation, and thus long‐term exposure to oxygen or water‐containing environments generally cause the decreased capability of MXene‐based devices. Thus, many experimental performance tests for MXenes are primarily conducted in nonaqueous electrolytes with sealed and rigid packages, which impede their practical utilization in flexible energy devices. Currently, only very few reports address the oxidation problem of MXenes via hydrophobic modifications and other methods. The adsorption of polyanionic groups on the edges of MXene nanoflakes can prevent their reactions with water molecules and prohibit MXenes from being oxidized. Additionally, high‐temperature hydrogen annealing and silylation treatment can also endow MXene nanoflakes with prominent antioxidative properties. Therefore, the proposition of reasonable strategies to design antioxidizant structures for MXenes will be a research direction that is worth deeply exploring.

Additionally, MXenes constitute the largest family of 2D materials, including more than 100 stoichiometric phases. However, only approximately 30 types of MXenes have been experimentally synthesized recently, and most of them are Ti‐based MXenes, which have been comprehensively investigated in energy disciplines. In recent years, other MXenes such as vanadium (V)‐based MXenes and niobium (Nb)‐based MXenes have demonstrated their unique features, which are beneficial to energy storage.^[^ ^8^, ^227^ ^]^ For instance, Nb_4_C_3_T*_x_* MXenes were demonstrated to possess better cyclic stability and rate performance.^[^ ^228^ ^]^ Also, V_2_CT*_x_* and V_4_C_3_T*_x_* MXenes expressed superior capacitances attributing to variable valence states of the V element and large interlayer distances.^[^ ^229^ ^]^ Thus, the development of new MXene phases is crucial for exploring and expanding their applications. Additionally, the application of MXene anodes for NLIBs has been extensively investigated and predicted by DFT calculations. The practical applications of these discussed MXenes and MXene‐based flexible materials in calcium‐ion batteries, magnesium‐ion batteries, aluminum‐ion batteries, potassium–oxygen batteries, calcium–oxygen batteries, lithium–carbon dioxide batteries, and so on should be urgently implemented.

In conclusion, it is imperative to address both the potential and the challenges associated with the industrial‐scale production of MXene‐based flexible energy storage devices. While numerous studies and evaluations have showcased the feasibility of large‐scale preparation, fabrication, and integration of MXene‐based materials and devices, a multitude of concerns remain unresolved.^[^ ^32^, ^49^, ^191^ ^]^ Transitioning from laboratory‐scale production to industrial settings prompts a series of crucial inquiries pertaining to reliability, automation, and societal implications. These encompass issues such as the yield ratio attainable in industrial production, the feasibility of automating the entire production process for MXene‐based flexible electronics, spanning synthesis to assembly, and the comprehensive assessment of environmental sustainability. Given the urgency and necessity of the matter at hand, it becomes imperative to seek answers to these questions through further in‐depth investigations.

The research evolution in these directions provides numerous opportunities for the further development of MXene‐based flexible materials. With more robust experimentation, it is anticipated that the aforementioned challenges of MXene‐based flexible energy electronics can be progressively overcome and a breakthrough of wearable energy devices will exist in the near future.

## Conflict of Interest

The authors declare no conflict of interest.
